# Bioinspired Ag/CeO_2_ and Ag/Bi_2_O_3_ nanohybrids synthesized with Nauplius graveolens for antioxidant, antibacterial, and insecticidal applications

**DOI:** 10.1038/s41598-026-42713-7

**Published:** 2026-04-19

**Authors:** Khaled M. Elattar, Mohammed S. El Hersh, Asma A. Al-Huqail, Giuliano Bonanomi, Ahmed M. Abd-ElGawad, Yasser A. El-Amier

**Affiliations:** 1 Unit of Genetic Engineering and Biotechnology, Mansoura University, El- Gomhoria St, Mansoura, 35516 Egypt; 2 Microbial Activity Unit, Department of Microbiology, Soils, Water and Environment Research Institute, Agricultural Research Center, Giza, 12619 Egypt; 3 Present Address: Chair of Climate Change, Environmental Development and Vegetation Cover, Department of Botany and Microbiology, College of Science, King Saud University, Riyadh, 11451 Saudi Arabia; 4 Department of Agricultural Sciences, University of Naples Federico II, Via Università 100, Portici, 80055 Italy; 5 Plant Production Department, College of Food & Agriculture Sciences, King Saud University, P.O. Box 2460, Riyadh, 11451 Saudi Arabia; 6 Botany Department, Faculty of Science, Mansoura University, Mansoura, 35516 Egypt

**Keywords:** *Nauplius graveolens*, Ag-doped metal oxide NCs, Antioxidant, Antimicrobial, Insecticidal activities, Biochemistry, Biological techniques, Biotechnology, Chemistry, Drug discovery, Microbiology, Plant sciences

## Abstract

A rising number of challenges of antibiotic resistance worldwide requires the creation of new antimicrobial and insecticidal agents. This incident stimulates a bioinspired methodology for the biosynthesis of novel and sustainable Ag/CeO_2_ and Ag/Bi_2_O_3_ nanohybrids. The high phytochemical contents of *Nauplius graveolens* extract, such as phenolics, flavonoids, and tannins, act as effective reducingand stabilizing agents for the synthesis of nanocomposites. *N. graveolens* extract and the synthesized nanohybrids also demonstrated a good antioxidant capacity; e.g., in DPPH assay, the extract had an IC_50_ of 0.067 mg/mL, whereas the IC_50_ of Ag/CeO_2_ and Ag/Bi_2_O_3_ NCs were 1.073 and 1.068 mg/mL, respectively. The reducible capacity by the FRAP assay is in line with the results of the DPPH assay. Notably, the nanohybrids exhibit a broad spectrum against different pathogenic bacterial species. The results of the insecticidal bioassays showed that both nanocomposites exhibited high dose-dependent insecticidal efficacy towards *Brevicoryne brassicae* and *Aphis craccivora*, with significantly lower values for the LC_50_ and significantly higher values for the toxicity index. The results indicated the novelty of these bioinspired nanohybrids as potential fighters against drug-resistant bacteria, as well as in the multifunctional effectiveness of antioxidant and insecticidal applications.

**Supplementary Information:**

The online version contains supplementary material available at 10.1038/s41598-026-42713-7.

## Introduction

Excessive use of antibiotics in clinical practice has encouraged the emergence and spread of resistant microbes, with an enormous burden on the economy and health of the world^1^. Presently, these resistant pathogens are becoming causative agents for infections that are increasingly difficult to treat, which translates into increased morbidity and mortality worldwide. Resistance arises through mechanisms in a wide range of forms, like enzymatic alteration of drugs, gene mutations altering bacterial targets, and efflux pumps for extrusion of antibiotics^2^. These adaptations have reduced the efficacy of traditional antibiotics, even for minor infections, and compromise food safety and industrial productivity^3^. Therefore, the quest for novel antimicrobial agents is an international priority.

Nanoparticles (NPs) represent a hopeful strategy to overcome resistance through multifaceted bactericidal mechanisms such as membrane disruption, intracellular interference, and oxidative stress^4^. Metal-based NPs, especially, offer the advantages of small size, large surface area, surface charge, and minimal chance of resistance^3^. Silver nanoparticles (AgNPs) have been most widely studied due to their broad-spectrum antimicrobial activity and limited resistance possibility, with mechanisms encompassing membrane damage, protein and DNA interaction, and reactive oxygen species (ROS) generation^5^.

Bimetallic nanostructures comprising two metals generally present greater antibacterial activity compared to the corresponding monometallic nanostructures^6^. Ag-Au, Ag-Cu, Fe-Ag, and Cu-Ni are a few systems that have manifested synergistic effects. For example, Ag/CeO_2_ nanocomposites have shown activity against *P. aeruginosa* and *S. aureus* with additional anticancer activity^7^, and Ag-Bi composites also manifest effective antibacterial activity^8^.

*Nauplius graveolens* (syn. *Astersicus graveolens*) is a medicinal plant rich in flavonoids, terpenoids, phenolics, and essential oils, comprising reducing and capping agents for green-mediated nanoparticle synthesis. Its extracts have been found to display antioxidant, antimicrobial, and pharmacological activities^9^, with phytochemical variation between plant organs making NPs have tunable properties^10^. Although Ag and SeO_2_ nanoparticle synthesis using this plant has been described^11^, the use of bimetallic nanocomposites such as Ag/CeO_2_ or Ag/Bi_2_O_3_ *via* green methods is comparatively less investigated^12^.

This study bridges this gap by using *N. graveolens* extract as a green reducing and stabilizing agent for Ag/CeO_2_ and Ag/Bi_2_O_3_ nanocomposite synthesis for the first time. Phytochemical profiling (GC-MS, total phenolics, flavonoids, tannins) was performed in parallel with antioxidant (DPPH, FRAP) and antibacterial assays. Structural and morphological features were examined by UV-Vis, XRD, HR-TEM, SEM, and EDX. The work demonstrates the dual bioactivity of these nanohybrids, providing an insight into the synthesis-structure-function relationship and potential as green therapeutic and antimicrobial agents.

## Materials and methods

### Reagents, materials and instruments

Analytical-grade reagents were purchased from Sigma Aldrich (USA), and PIOCHEM laboratory chemicals with a high degree of purity (for details see Supplementary file, **Section S1**). *Nauplius graveolens* shoots were collected from Wadi El-Rashrash, Northeastern Desert, Egypt (29°26′21.32″N, 31°26′2.62″E). The collection site is not a protected area, and no specific permits or licenses were required for the collection of this wild plant species. The plant was identified based on standard floristic keys^13^ and further confirmed using Plants of the World Online (POWO, Royal Botanic Gardens, Kew)^13^. A voucher specimen was prepared and deposited in the Herbarium of the Botany Department, Faculty of Science, Mansoura University, Egypt (Voucher No. Mans. 001019007003). The plant material used in this study was formally identified and authenticated by **Dr. Yasser A. El-Amier**, Assistant Professor of Plant Ecology, Faculty of Science, Mansoura University, Egypt. The identification of *Nauplius graveolens* (Forssk.) Wikstr. was carried out using standard taxonomic keys and relevant floristic references. The authenticated plant material was subsequently used for the preparation of the aqueous extract applied in the green synthesis of Ag/CeO_2_ and Ag/Bi_2_O_3_ nanohybrids.

UV-Vis spectroscopy was employed on Spekol 11, Analytic Jena, Germany, SEM on FEI, Czech, FTIR analysis on Thermo-Fisher Nicolet IS10 Spectrophotometer and HR-TEM on Thermo Scientific Talos F200i. Zeta potential measurements were performed on a HORIBA Scientific SZ-100 “Ver 2.40” instrument. XRD patterns were recorded using a Pan Analytical Philips system. GC-MS analysis was performed on a Trace GC-TSQ mass spectrometer (Thermo Scientific, Austin, TX, USA) (see Supplementary file, **Section S1**).

### Extraction of *N. graveolens* shoots

*Nauplius graveolens* shoots were collected, washed, and air-dried. A total of 10 g of dried plant material was accurately weighed and mixed with 100 mL of 70% ethanol solution, following the method described in previous studies^14^. The mixture was placed in a conical flask and incubated in a horizontal water bath shaker (Memmert WB14, Schwabach, Germany) at 220 rpm and 40 °C for 2 h. After incubation, the mixture was allowed to cool to room temperature and then left to soak overnight. The resulting extract was filtered using Whatman filter paper and used immediately for nanocomposite synthesis, phytochemical screening, and spectroscopic analysis. If needed, the extract could be stored in a refrigerator for up to one week without significant loss of activity.

### Green synthesis of nanocomposites

A 100 mL solution of silver nitrate (10 mM) was prepared in deionized water and stirred at room temperature. In this silver nitrate solution, 100 mL of *N. graveolens* extract (6.78 mg/mL) was added dropwise under stirring. In order to synthesize Ag/CeO_2_ NC, 30 mL of cerium oxide suspension (5 mM) wasadded to 70 mL of the previously prepared AgNPs solution under stirring. Similarly, Ag/Bi_2_O_3_ NC was synthesized by adding 30 mL of bismuth oxide suspension (5 mM) into 70 mL of the Ag NP solution. Both reaction mixtures were stirred while gradually increasing the temperature to 60–70 °C and maintained for 5 h. Then, the mixtures were sonicated at 85 °C for 2 h. The resultant solid nanomaterials were centrifuged at 10,000 rpm for 10 min. The pellets obtained were washed with ethanol and deionized water several times to remove residual organic groups and unreacted precursors. The nanocomposites were dried at 100 °C in a hot air oven and were characterized using SEM and XRD analyses^15^.

To check the reproducibility and reliability of the results, three separate batches of Ag/CeO_2_ and Ag/Bi_2_O_3_ nanocomposites were synthesized under identical reaction conditions. The results from the XRD patterns showed that the position of the peaks and intensity for all three batches varied by less than 5%. The FTIR results also confirmed the presence of identical functional groups in all three batches. The SEM results also confirmed identical morphology for all three batches.

Although the present study does not comprise a comparative analysis of the synthesized nanoparticles with commercially available nanoparticles, the findings of this study are indicative of the fact that the green synthesized nanohybrids have reproducible structural and morphological properties. Future studies may compare the performance of these nanocomposites with commercially available nanocomposites.

### Phytochemical analysis

The contents of tannins, total phenolics (TPC), and total flavonoids (TFC) in the *N. graveolens* extract and nanocomposite solutions were measured through standard colorimetric assays. Tannin content was ascertained using vanillin-hydrochloride assay^16^, where a reagent was prepared by combining 30% hydrochloric acid with 4% vanillin in methanol. TPC was assessed by Folin-Ciocalteu assay^16^ through adding 100 µL of every sample to a solution containing 1:10 diluted Folin-Ciocalteu reagent and sodium carbonate. TFC was investigated by aluminum chloride assay^16^ through adding 0.3 mL of 5% sodium nitrite and 0.3 mL of 10% aluminum chloride to 100 µL of each sample dissolved in distilled water. Additional experimental details are provided in supplementary information (**Section S1**).

### Antioxidant activity

#### DPPH assay

Antioxidant activity of the samples was measured by DPPH• assay using ascorbic acid as a standard17. The absorbance of each solution after incubation was measured at 517 nm. A positive control was a combination of an equal volume of DPPH• solution and methanol, in which methanol was used as a negative control. The remaining percentage of DPPH• radicals was calculated using the given equation (Eq. 1):

1 $$\mathrm{\%}\mathrm{R}\mathrm{e}\mathrm{m}\mathrm{a}\mathrm{i}\mathrm{n}\mathrm{i}\mathrm{n}\mathrm{g}\mathrm{D}\mathrm{P}\mathrm{P}\mathrm{H}\mathrm{r}\mathrm{a}\mathrm{d}\mathrm{i}\mathrm{c}\mathrm{a}\mathrm{l}\mathrm{s}=\frac{\left[\mathrm{D}\mathrm{P}\mathrm{P}\mathrm{H}{\cdot}\right]\mathrm{T}}{\left[\mathrm{D}\mathrm{P}\mathrm{P}\mathrm{H}{\cdot}\right]\mathrm{T}=0}\mathrm{x}100$$

IC_50_ values are expressed in mg/mL ± SD, and were calculated from this curve, which is the concentration required for 50% scavenging of the DPPH^•^ radicals (**Section S1**).

#### Ferric-reducing power assay

The plant extract and nanocomposites’ capacity to reduce was examined^18^. In short, the test sample (1 mL) was mixed with phosphate buffer solution (2.5 mL, 0.2 M, pH 6.6) and potassium ferrocyanide (2.5 mL, 1%). The solution was heated at 50 °C for 20 min. After incubation, 2.5 mL of 10% trichloroacetic acid was added to the mixture and centrifuged at 3000 rpm for 10 min. Then, a 2.5 mL aliquot of the resulting supernatant was combined with 2.5 mL distilled water and 0.5 mL ferric chloride solution (0.1%). The sample was measured for its absorbance at 700 nm. Reducing powers of the samples were also compared to those of the standard ascorbic acid (**Section S1**).

### Agar well diffusion method

To determine the antibacterial activity of the plant extract and synthesized nanocomposites, an agar well diffusion method was employed against eight bacterial pathogens, following a standard protocol^19^. Samples were tested at concentrations of 6.78 mg/mL for nanocomposites and extract. Fresh bacterial cultures were prepared and diluted to a density of 1–2 × 10^8^ CFU/mL, which is equivalent to a 0.5 McFarland standard. Aseptically, 9 mm diameter wells were made on the agar using a sterile cork borer. 100 µL of test sample at the necessary concentration was added to the wells. Incubation was carried out for 24 h at 37 °C. Following incubation, the antibacterial activity was examined by measuring the diameter of the inhibition zones around each well.

The bacterial strains used in this study were obtained from recognized culture collections, and their accession numbers are listed as follows: *Escherichia coli* (ATCC 10536), *Salmonella typhimurium* (ATCC 25566), *Klebsiella pneumoniae* (ATCC 10031), *Enterobacter cloacae* (DMS 30054), *Bacillus subtilis* (DMS 1088), *Bacillus cereus* (EMCC 1080), *Staphylococcus aureus* (ATCC 6538), and *Staphylococcus epidermidis* (EMCC 1353t).

### Insecticidal assay

#### Colony maintenance

Colonies of *Aphis craccivora* and *Brevicoryne brassicae* were collected from the experimental farm of the Faculty of Agriculture, Mansoura University, Egypt. The population of insects was inspected for ensuring insecticide-free. Cowpea (*Vigna unguiculata*) plants were used as host material in case of *(A) craccivora*, while cabbage (*Brassica oleracea* var. *capitata*) served as host for *(B) brassicae*. Colonies were maintained under laboratory conditions in a plastic house (2.5 × 2.5 × 2.0 m). As these insect populations were field-collected, they are not part of any formal culture collection and therefore do not have accession numbers; this has been clarified for transparency.

Laboratory colonies of *Aphis craccivora* and *Brevicoryne brassicae* are maintained on their respective host plants under controlled environments at the Faculty of Agriculture, Mansoura University. These populations, although not officially deposited in culture collections, are from field-collected populations, and molecular identification has been carried out using mitochondrial cytochrome oxidase I (COI) gene sequence information. The reference sequence information for a representative sequence of (A) craccivora is available in GenBank under accession number KT889380. In addition, reference sequence information for the mitochondrial genome is available for *(B) brassicae* and can be used as standard sequence information for molecular identification and confirmation of species identity.

#### Bioassay procedure

Insecticidal activity of *N. graveolens* extract against *(A) craccivora* on cowpea leaves and *(B) brassicae* on cabbage leaves was assayed using the leaf-spray method. Five varying concentrations of plant extract (50–250 ppm) and nanocomposite preparations (5-100 ppm) were prepared in distilled water with 0.1% Tween-80 as the surfactant. For each concentration, thirty aphids were treated with cowpea or cabbage leaf discs (5.0 cm diameter) in Petri dishes containing 1.5% agar. Each treatment included the spraying of 2 mL test solution on aphids, with three replications for each concentration. Control groups included the spraying of distilled water and Tween-80 only. Later, insects were incubated under room temperature, and mortality was assessed after 24 h.

#### Data Analysis

Observed mortality was corrected with Abbott’s formula (Eq. 2)^20^:

2 $$CorrectedMortality\left(\%\right)=\frac{Mortalityintreatment-Mortalityincontrol}{100-Mortalityincontrol}\times100$$

Concentration-mortality responses were analyzed according to Finney’s probit analysis technique^21^ for the calculation of LC_50_ and LC_90_ values at 95% confidence limits and slopes of regression lines (LC-P lines). The toxicity index was calculated based on Sun’s equation (Eq. 3)^22^:

3 $$ToxicityIndex=\frac{{LC}_{50}\left(reference\right)}{{LC}_{50}\left(treatment\right)}\times100$$

where azadirachtin was used as a reference standard.

### Statistical analysis

All results were expressed as the mean of three independent replicates and reported as mean ± standard deviation (SD). Statistical analysis was performed using the Statistical Package for the Social Sciences (SPSS), version 21. Differences between groups were considered statistically significant at a p-value ≤ 0.05. For toxicity tests, LC_50_ and LC_90_ values were obtained through probit analysis. The 95% confidence intervals for these values were also obtained to evaluate the reliability of the values. The addition of 95% confidence intervals gives an idea of the variability for any set of values. Moreover, it makes the results obtained for biological activity statistically sound. The use of replicate values also ensures that the differences obtained are reliable.**3. Results and Discussion**.

### GC/MS mass spectroscopy

**Fig. 1:**
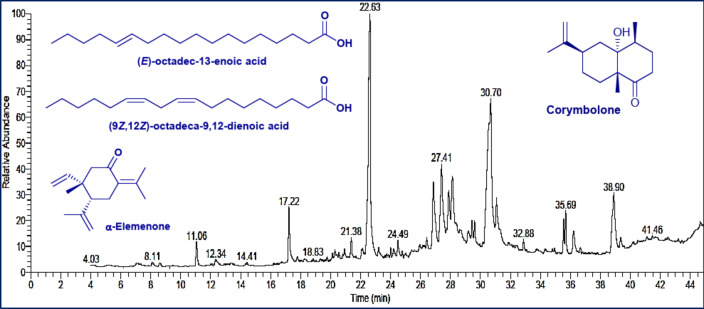
Chromatogram of the basic volatile components extracted from *N. graveolens* by GC-MS.

The GC/MS analysis result revealed that the chemical composition of the volatile components extracted from *N. graveolens* was very heterogeneous (Fig. 1**& Table ****S1**). Among all identified compounds, Corymbolone, a sesquiterpene, was the major one, comprising 20.11% of the total composition, followed by the fatty acid (*E*)-octadec-13-enoic acid, present at 11.53%. The established volatile compounds in *N. graveolens* had a complex chemical profile of sesquiterpenes (33.72%), fatty acids (30.23%), and lipids (25.67%) that comprised nearly 90% of the total volatiles (**Figure ****S1**).

GC-MS analysis of *N. graveolens*-based nanocomposite showed volatile profile rich in bioactive compounds, with *cis*-chrysanthenyl acetate, 1,8-cineole, camphor, and borneol being the major components. In comparison with the findings reported by Alilou et al.^23^., our GC-MS analysis of *Asteriscus graveolens* essential oil depicts similarities but exemplary differences in terms of chemical composition. The subtle compositional differences may be due to geographical, climatic, or seasonal factors affecting the biosynthesis of the secondary metabolites. Despite such variations, the dominance of oxygenated monoterpenes as the major chemical class in both research studies indicates the essential oil’s-maintained antioxidant and antifungal activity across different populations of *A. graveolens*.

### Mechanism of nanocomposites’ formation

**Fig. 2:**
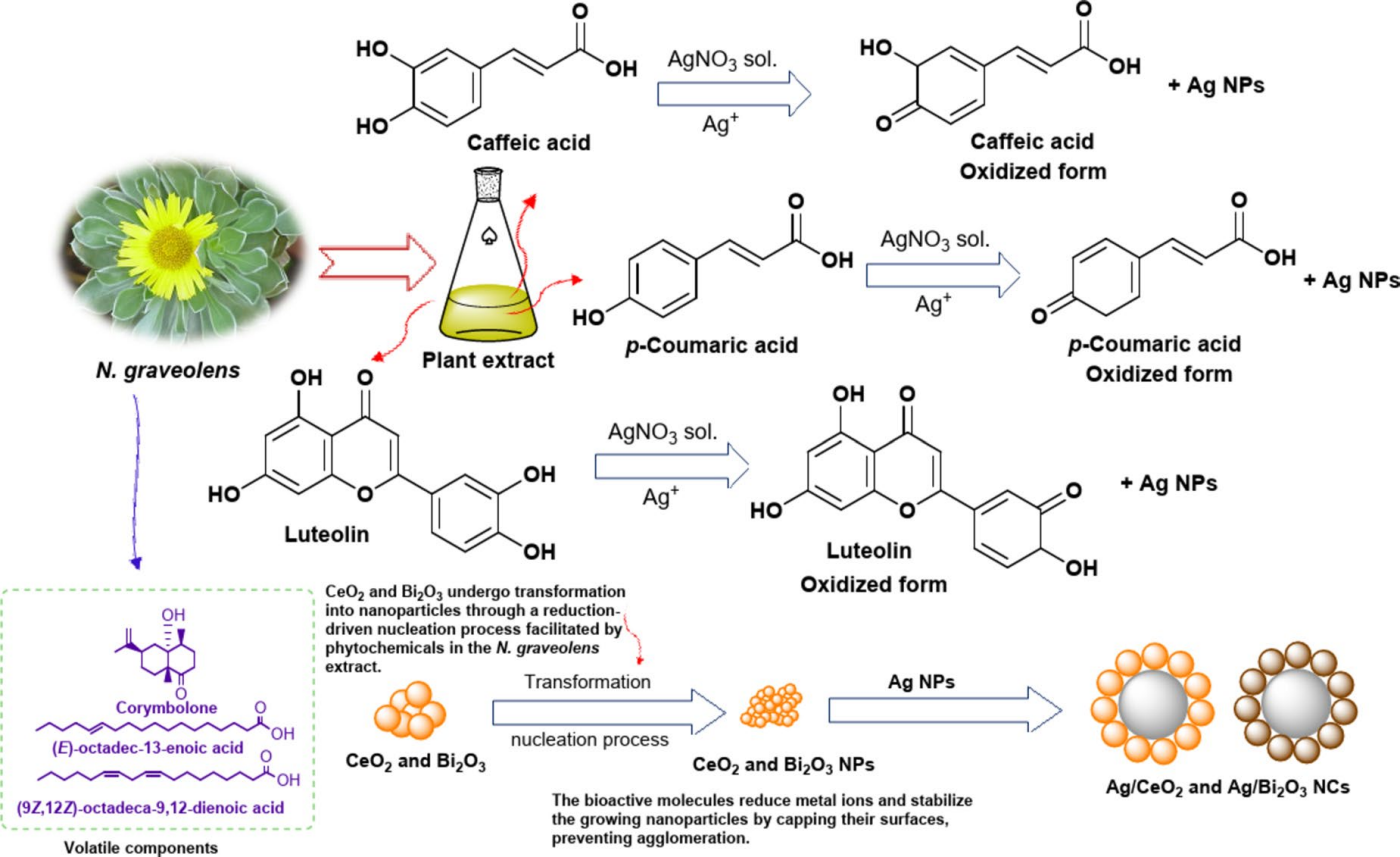
The conceivable mechanism for the nanocomposites’ formation.

The mechanism for the green synthesis of Ag/CeO_2_ and Ag/Bi_2_O_3_ NCs from *N. graveolens* extract is proposed through the active role of phytochemicals, particularly phenolics, flavonoids, and tannins^24^, in the reduction, nucleation, and stabilization processes (Fig. 2). When the plant extract is combined with silver nitrate solution, bioactive molecules present in the extract donate electrons to Ag^+^ ions, which are consequently reduced to metallic silver (Ag^0^)^25^. Phenolic hydroxyl groups are particularly valuable as reducing agents in terms of electron donation, while flavonoids and tannins stabilize the resultant Ag nanoparticles through surface adsorption and steric blocking to prevent agglomeration^26^. On adding cerium oxide or bismuth oxide into the Ag NPs dispersion, bio-reducing agents trigger interactions between Ag NPs and metal oxide surfaces to enable nucleation of Ag onto CeO_2_ and Bi_2_O_3_ matrices^27^. The chelating ability of polyphenols and tannins also supports the anchoring and dispersion of the silver particles on the oxide surfaces, leading to homogeneous and stable nanocomposites^28^.

The role of specific volatile compounds in the green synthesis of nanocomposites is very significant^29^. The presence of hydroxyl and carbonyl groups in corymbolone makes it an electron donor, which reduces Ag^+^, Ce^4+^, and Bi^3+^ ions. Unsaturated fatty acids such as (*E*)-octadec-13-enoic acid and linoleic acid coordinate with metal surfaces through their carboxyl groups, which act as mild reducing agents and capping agents to prevent aggregation of nanoparticles^30^. The presence of double bonds in fatty acids further increases the electron delocalization process, which is very effective in the reduction process during nucleation^31^. In these processes, the phenolic and terpenoid compounds are oxidized to quinones or ketones, transferring electrons to the metal ions. The unsaturated fatty acids also help in delocalizing electrons in the double bonds of the fatty acids, making the radical species more stable and efficient in reduction reactions. These processes help in the simultaneous formation of nanoparticles and the prevention of aggregation by the adsorption of the oxidized phytochemicals on the surface of the nanocomposites. The combination of phenolics, flavonoids, tannins, and other volatile terpenoids makes the process of reduction, nucleation, and stabilization very effective, resulting in the formation of homogeneously dispersed and stable Ag/CeO_2_ and Ag/Bi_2_O_3_ nanocomposites.

### Characterization of nanocomposites

#### FTIR spectroscopy

FTIR analysis of *N. graveolens* extract and derived nanocomposites Ag/CeO_2_ NC and Ag/Bi_2_O_3_ NC delivered essential data about functional groups that participated in nanocomposite synthesis (Fig. 4a **& Table ****S2**). Two absorption bands in the analysis of *N. graveolens* extract at 1631 cm^− 1^ and 1598 cm^− 1^ represent the aromatic rings which probably originate from phenolic compounds. The aromatic characteristics of C = C stretch at 1515 cm^− 1^ and C-H bending at 1457 cm^− 1^ show that the structural component includes aromatic properties. The functional groups detected in the analysis match the active role these groups have ingreen-based metal nanocomposite synthesis.

**Fig. 3:**
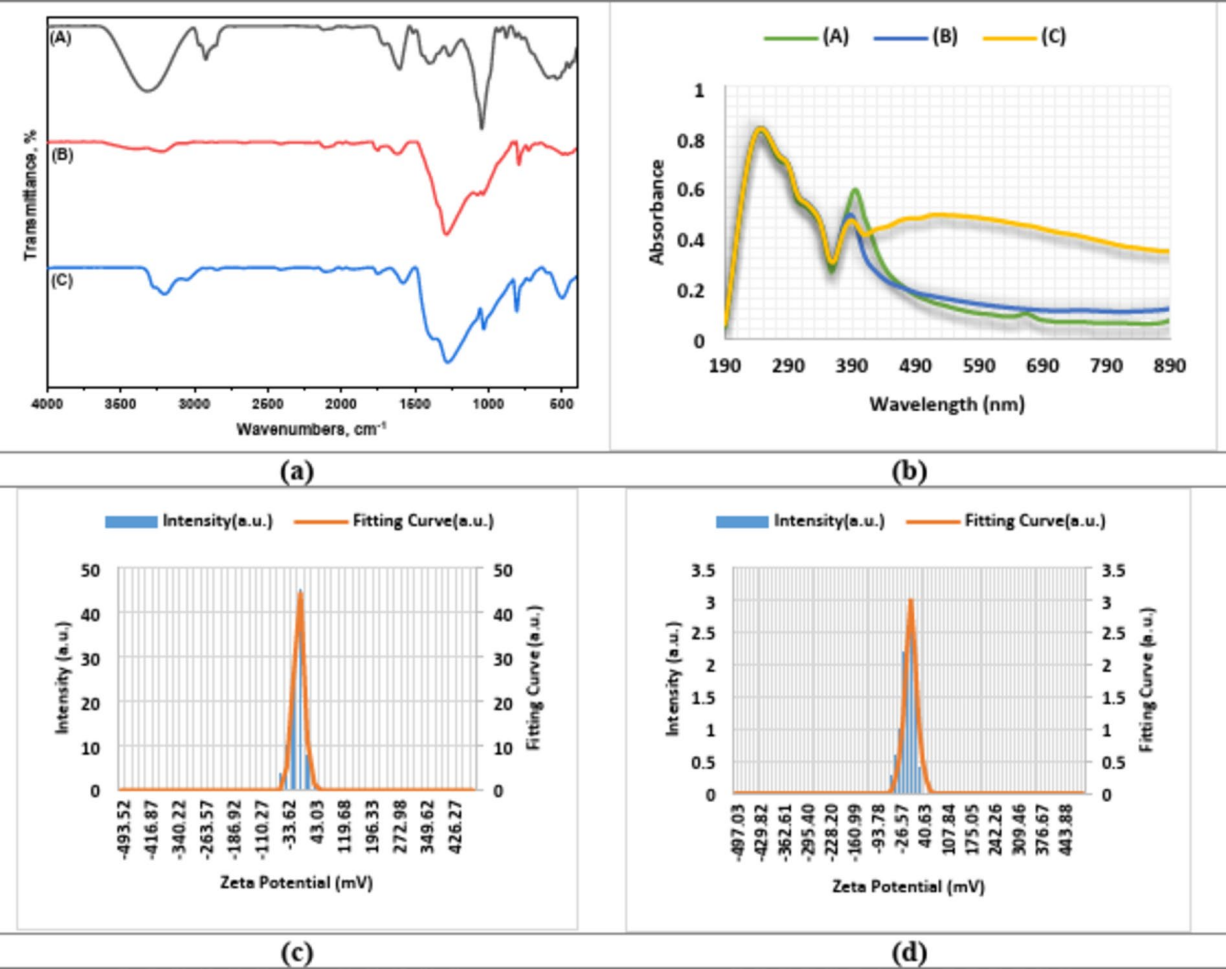
FTIR, UV-visible spectroscopy, and zeta potential analysis of *N. graveolens extract* and nanocomposites. (a) FTIR spectral analysis of *N. graveolens* extract (A) and its derived Ag/CeO_2_ NC (B) and Ag/Bi_2_O_3_ NC (C). (b) UV-visible spectroscopy at varied wavelengths (nm). (A) Referred to *N. graveolens* extract; (B) Referred to Ag/CeO_2_ NC; (C) Referred to Ag/Bi_2_O_3_ NC. (c) Zeta potential analyses of Ag/CeO_2_ NC. (d) Zeta potential analyses of Ag/Bi_2_O_3_ NC.

Characteristic absorption bands for the Ag/CeO_2_ NC spectrum showed 3243 cm^− 1^ (O-H stretch), 3191 cm^− 1^ (O-H stretch), and 1773 cm^− 1^ (C = O stretch), suggesting the presence of hydroxyl and carbonyl groups due to the extract of *N. graveolens*. The lower wavenumber bands reflect the metal oxide stretch, especially the absorption bands at 501 and 473 cm^− 1^, which are assigned to Ce-O vibrations and confirm the successful incorporation of CeO_2_ within the nanocomposite. Key absorption bands for the Ag/Bi_2_O_3_ NC spectrum revealed bands at 3285 cm^− 1^ for O-H stretching, 3193 cm^− 1^ for O-H stretching, and 3048 cm^− 1^ for C-H stretching. The lower wavenumber region reflects the Bi-O bond, with characteristic peaks at 606 and 497 cm^− 1^, confirming the formation of the Bi_2_O_3_ component in the nanocomposite. The FTIR spectra of Ag/CeO_2_ NC and Ag/Bi_2_O_3_ NC showed similar groups of functional groups, including hydroxyl, carbonyl, and aromatic compounds, which originated from the *N. graveolens* extract, probably playing the main role in the reduction of the metal ions and in the stabilization of the metal oxide nanoparticles.

Apart from this purpose of using them in the synthesis of compounds, the surface functional groups can also be exploited for the purpose of biological activity by the augmentation of electrostatic interactions with the bacterial cell membrane and the promotion of ROS production. However, it is important to note that FTIR is a semi-qualitative technique and cannot be employed for the quantitative determination of surface elemental composition and oxidation states. A further study of the surface chemistry is required for this purpose, such as XPS analysis, for the quantitative determination of certain surface functional groups and metal oxidation states, and their association with biological activity. This study is planned for the future to gain further insight into this issue.

The FTIR spectra of both the *N. graveolens* extract and its corresponding nanocomposites, Ag/CeO_2_ NC and Ag/Bi_2_O_3_ NC, confirm the involvement of plant-derived functional groups in the synthesis and stabilization of the nanoparticles. Both extracts and nanocomposites present hydroxyl, carbonyl, and aromatic groups, proving a dual role for *N. graveolens* extract as both a reducing agent and a stabilizer of the nanoparticles.

#### UV-visible spectroscopy

Figure 3b shows the UV-Vis spectra of N. graveolens extract, Ag/CeO_2_, and Ag/Bi_2_O_3_ NCs, illustrating their optical properties and confirming nanocomposite formation (**Table S3**). The extract exhibited a strong peak at 398 nm (0.521) owing to phytochemicals, which acted as reducing and stabilizing agents. Ag/CeO_2_ showed a blue-shifted peak at 385 nm (0.402), confirming composite formation. Ag/Bi_2_O_3_ exhibited peaks at 389 nm (0.379) and 519 nm (0.430), the second one due to the surface plasmon resonance (SPR) of Ag nanoparticles within the Bi_2_O_3_ matrix.

These spectra are characteristic of typical π→π* and n→π* transitions of aromatic and heteroatom-containing compounds. Blue shifts in Ag/CeO_2_ and Ag/Bi_2_O_3_ reflect metal oxide-plant constituent interactions, while the new 519 nm band in Ag/Bi_2_O_3_ is characteristic of SPR and charge transfer interactions. Together, the shifts and transitions here confirm nanocomposite formation and altered optical properties through metal incorporation.

#### Zeta potential analysis

A variation of zeta potential was employed to determine the surface charge and colloidal stability of the nanocomposites (Figs. 3c, d & S2). Ag/CeO_2_ NC was − 0.000005 cm^2^/Vs, which equated to an average of -0.6 mV zeta potential (Fig. 3c). On the other hand, the Ag/Bi_2_O_3_ nanocomposite showed the average zeta potential at 0.7 mV with a corresponding electrophoretic mobility of 0.000054 cm^2^/Vs (Fig. 3d). The Ag/Bi_2_O_3_ NC surface positive charge indicates better dispersion in suspension state compared to Ag/CeO_2_ NC. Negi et al.^32^. established that Ag/CeO_2_ nanostructures possessed a zeta potential of about − 12.5 mV, attributing the negative charge to oxygen vacancies on the surface and to Ce^3+^/Ce^4+^ redox couples that guarantee electrostatic repulsion and stability in dispersion. For Ag/Bi_2_O_3_ systems, Zhao et al.^33^ reported a zeta potential of + 15 to + 22 mV, which is much greater than our obtained value of + 7.0 mV. Their study attributed the favorable surface charge to Bi^3+^ ions and enhanced interfacial charge separation in Ag/Bi_2_O_3_ heterostructures.

The relatively low values of zeta potentials calculated in this work, which are close to neutrality, indicate a lack of significant electrostatic stabilization, since values greater than ± 30 mV are considered characteristic of good stability. Thus, it is believed that the stability of the dispersions of the synthesized nanocomposites is not only due to electrostatic repulsion but possibly to steric hindrance, which is due to the presence of a capping layer from phytochemicals adsorbed on the nanoparticle surfaces.

The presence of these compounds could result in steric hindrance that could reduce the rate of direct contact between the nanoparticles, thereby acting as an impediment to the low surface charge. The low surface charge could result in the slow aggregation of the nanoparticles due to the near-neutral zeta potential, especially after prolonged periods of storage. The aggregation could impact the surface area and possibly the activity of the nanoparticles. Although there is a possibility of activity in moderately aggregated nanoparticles, this does not mean that surface-functionalized nanoparticles cannot effectively interact with microbial or cellular targets; therefore, optimization of surface charge could enhance the colloidal stability of the nanoparticles for longer periods of application. Briefly, our Ag/CeO_2_ and Ag/Bi_2_O_3_ NCs would have values of zeta potential that are lower than the results of literature, measured results are comparable and point towards the controlled dispersion behavior and the ability to interact with biological or environmental targets *via* the barrier of surface charges.

#### High-resolution transmission electron microscopy (HR-TEM)

**Fig. 4:**
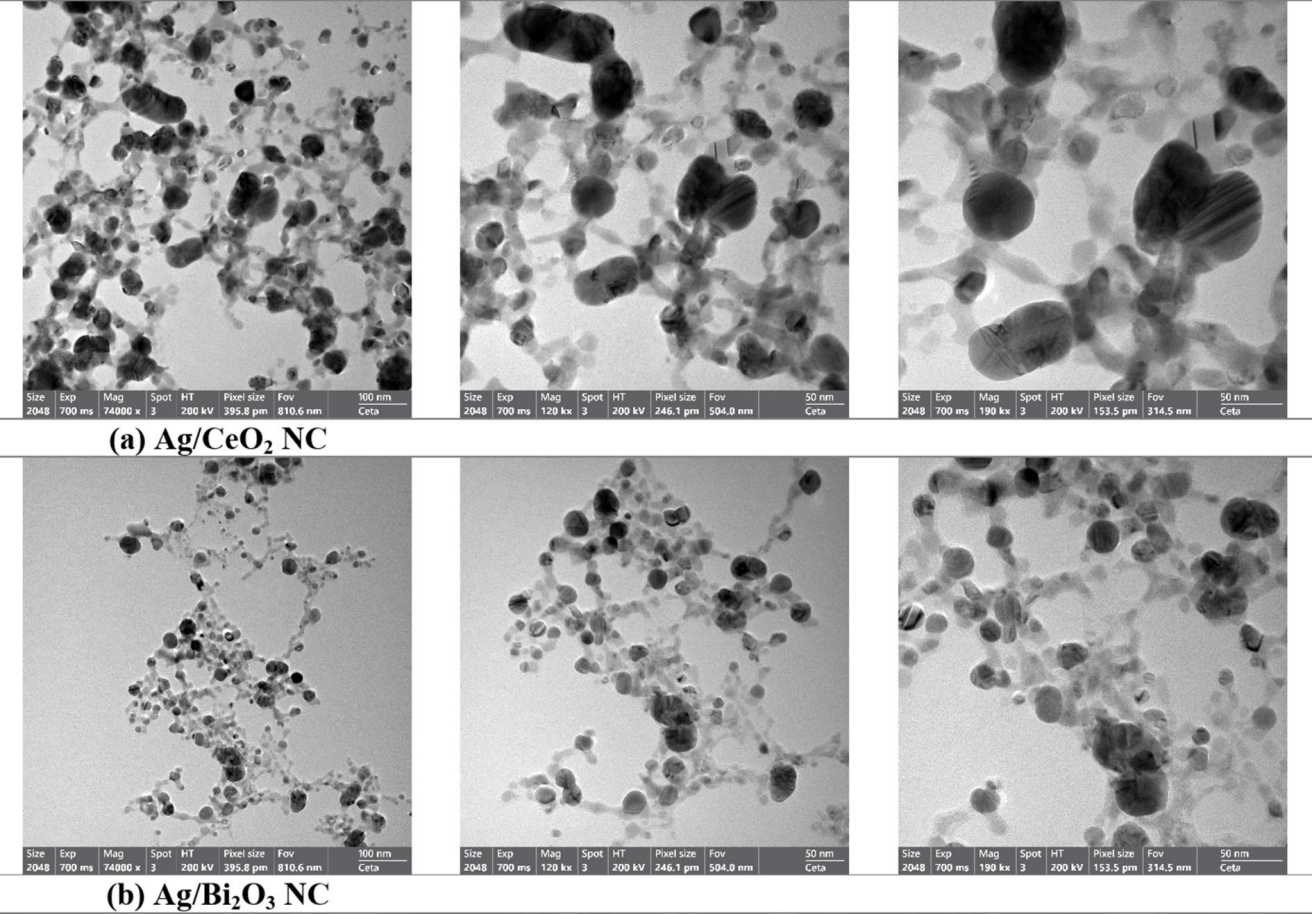
HR-TEM micrographs of (a) Ag/CeO_2_ NC (Exposure: 700 ms; Magnification (Mag): 74000 x; HT (High Tension): 200 KV; Pixel Size: 395.8 pm; FOV (Field of View): 810.6 nm) and (b) Ag/Bi_2_O_3_ NC (Exposure: 700 ms; Magnification (Mag): 74000 x; HT (High Tension): 200 KV; Pixel Size: 395.8 pm; FOV (Field of View): 810.6 nm). (a) Ag/CeO2 NC, (b) Ag/Bi2O3 NC.

The structural morphology of Ag/CeO_2_ NC, as was identified from HR-TEM micrographs (Fig. 4a), is spherical and irregular nanoparticles of hybrid morphology. The bright and less bright areas are smaller Ag (5–15 nm) and bigger CeO_2_ (20–40 nm) nanoparticles, respectively. Ag nanoparticles are homogeneously dispersed on the CeO_2_ matrix, creating Ag/CeO_2_ interfaces that lead to high catalytic activity and electron transfer.

The HR-TEM micrograph (Fig. 4b) for Ag/Bi_2_O_3_ nanocomposite is also similar, with evident, dark spherical or slightly irregular Ag nanoparticles well dispersed on a light Bi_2_O_3_ matrix with minor agglomeration. Lattice fringes mark the crystalline nature of Ag, while amorphous and crystalline phases are present in the Bi_2_O_3_ matrix. Nanoscale dimensions, uniform distribution, and interfacial contact reflect synergistic strong interactions with high electron mobility and oxygen activation. These structural properties agree with earlier research by David et al.^34^. HR.TEM analysis verifies the formation of Ag/CeO_2_ and Ag/Bi_2_O_3_ NCs successfully with well-dispersed Ag nanoparticles and strong interfacial interactions necessary for their functional and antibacterial properties.

#### Scanning electron microscopy (SEM) and EDX analysis

**Fig. 5:**
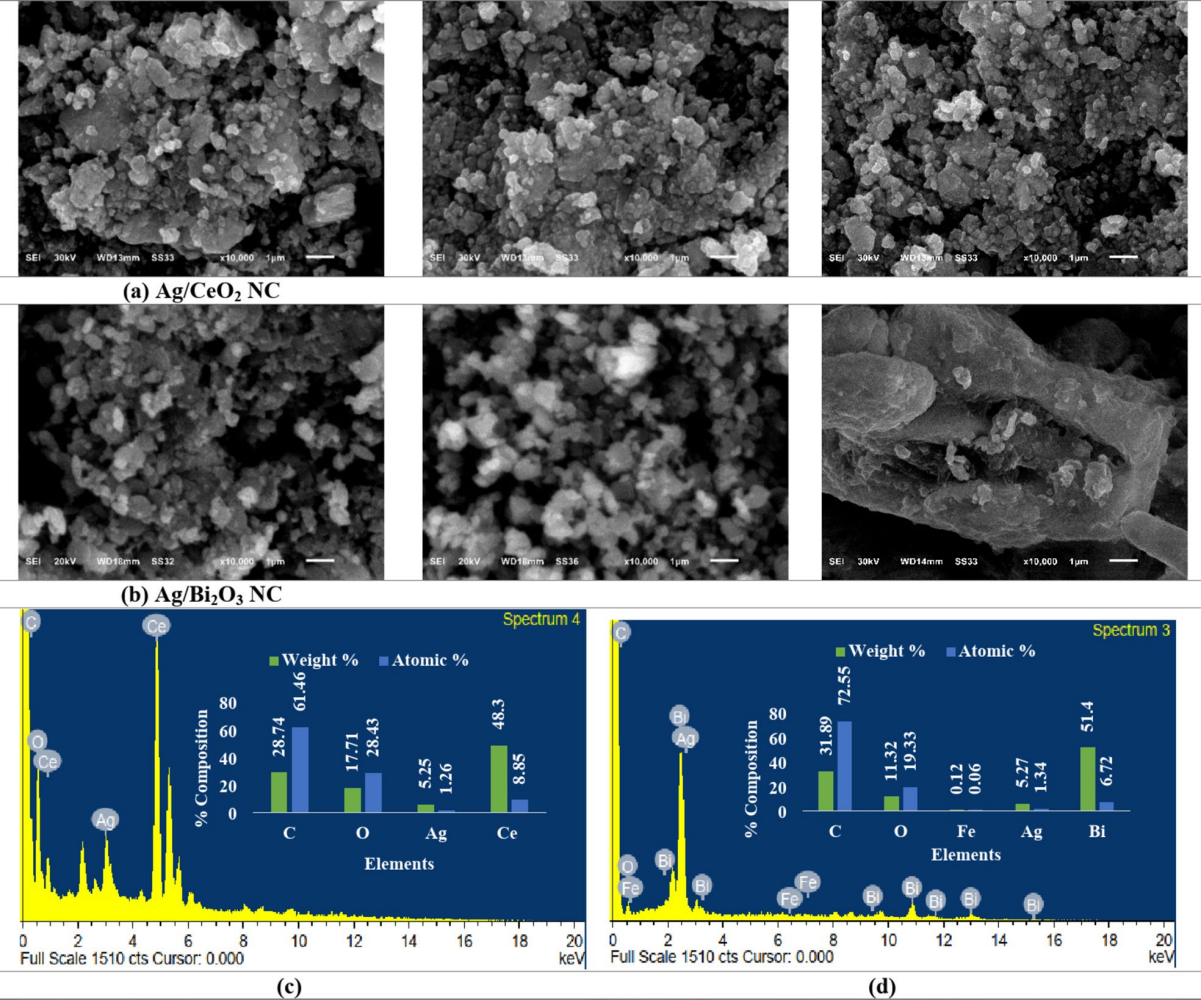
(a) SEM micrographs of Ag/CeO_2_ NC (captured at 30 KV, with a working distance (WD) of 13 mm, and magnified 10,000 times (indicated by a 1 μm scale bar)). (b) SEM micrographs of Ag/Bi_2_O_3_ NCs (captured at 20 KV with a working distance (WD) of 18 mm and magnified 10,000 times (indicated by a 1 μm scale bar)). (c) EDX analysis of Ag/CeO_2_ NC, and (d) EDX analysis of Ag/Bi_2_O_3_ NC. (a) Ag/CeO2 NC, (b) Ag/Bi2O3 NC.

SEM micrographs (Figs. 5a, b) reveal characteristic morphological differences between the two nanocomposites. Ag/CeO_2_ reveals aggregated morphology with irregular shapes, where smaller sphere nanoparticles are embedded in a porous ceria matrix (Fig. 5a). Contrast between the Ag as well as CeO_2_ regions observed in the results hints towards uniform incorporation. In contrast, Ag/Bi_2_O_3_ appears as an explicit composite structure with bimodal particle distribution, where smaller Ag nanoparticles are uniformly decorated over larger Bi_2_O_3_ nanoparticles, evidence for strong interfacial bonding with an effective composite structure (Fig. 5b).

EDX spectra confirmed the successful incorporation of silver in both CeO_2_ and Bi_2_O_3_ nanocomposite matrices (Figs. 5c, d). In the Ag/CeO_2_ NC, elemental composition totaled predominantly cerium (48.3 wt%, 8.85 at%) and oxygen (17.71 wt%, 28.43 at%) in accordance with the CeO_2_ lattice, where silver was unequivocally found at 5.25 wt% (1.26 at%), substantiating its effective loadings and distribution in the oxide support. Relative carbon (28.74 wt%, 61.46 at%) content is attributed to the phytochemicals of the *N. graveolens* extract acting as the cap-and-stabilizing species, in addition to the conductive carbon film used during SEM-EDX determinations. In the Ag/Bi_2_O_3_ NC counterpart, the predominant constituent was bismuth (51.4 wt%, 6.72 at%), with dominant oxygen (11.32 wt%, 19.33 at%) and silver (5.27 wt%, 1.34 at%), substantiating uniform silver decoration on the Bi_2_O_3_ support. Compared to Ag/CeO_2_, the Ag/Bi_2_O_3_ NC expressed slightly higher silver inclusion with high bismuth enrichment, accrediting successful nanocomposite construction with tight metal-oxide integration. Detection of high carbon in the respective systems also ensures the dual role of the plant metabolites in stabilizing nanoparticles and preventing uncontrolled aggregation. Generally, the EDX profiles substantiate the SEM findings by confirming the nanoscale coexistence between the Ag with the CeO_2_ or Bi_2_O_3_.

#### X-ray diffraction (XRD)

**Fig. 6:**
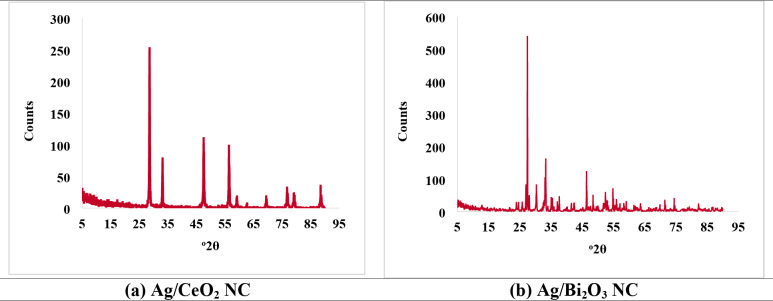
X-ray diffraction (XRD) patterns of (a) Ag/CeO_2_ NC and (b) Ag/Bi_2_O_3_ NC. The Ag/CeO_2_ NC shows characteristic peaks at 2θ = 28.62°, 33.19°, 47.54°, 56.39°, and 59.22° corresponding to the (111), (200), (220), (311), and (222) planes of cubic CeO_2_, confirming high crystallinity. The Ag/Bi_2_O_3_ NC exhibits sharp peaks at 2θ = 27.45°, 31.85°, 32.91°, and 46.84° corresponding to the (120), (101), (200), and (311) planes of Bi_2_O_3_, indicating successful formation of the crystalline nanocomposite. The absence of distinct Ag peaks suggests well-dispersed silver nanoparticles within the metal oxide matrices.

The XRD pattern of Ag/CeO_2_ NC (Fig. 6a **& Table S4**) exhibited a very sharp peak at 2θ = 28.6204^ο^ with a count intensity of 249.88 counts, along with having an FWHM of 0.0984^ο^, since it is reflecting the (111) plane of cubic CeO_2_. The XRD pattern had peaks at 33.1871^ο^ (71.20 counts, 0.1574^ο^ FWHM), 47.5360° (92.92 counts, 0.2755^ο^ FWHM) and 56.3943^ο^ (85.01 counts, 0.1968° FWHM) and these were at 2θ = 59.2163° (13.04 counts, 0.3149^ο^ FWHM) for CeO_2_ planes (200), (220), (311) and (222), respectively. The most intense peak at 28.6204^ο^ 2θ (100% relative intensity) reflects the dominant crystallographic orientation of the CeO_2_ phase.

Alternatively, the XRD pattern of the Ag/Bi_2_O_3_ NC (Fig. 6b **& Table S5**) showed several sharp peaks because of the specific planes of diffraction. The highest peak was found at 2θ = 27.4492^ο^. The intensity associated with this is 534.99 counts, having a FWHM of 0.1181^ο^. Consistently, for Bi_2_O_3_, it is the (120) plane that has a relative intensity of 100% and corresponds to the strongest peak. The obtained data confirmed the presence of crystalline Bi_2_O_3_ in the nanocomposite, the most intense plane being at (120). The XRD analysis confirms the successful synthesis of highly crystalline Ag/CeO_2_ and Ag/Bi_2_O_3_ nanocomposites, with well-defined peaks and nanoscale crystallite sizes supporting their potential in functional applications.

Moreover, the observed XRD peaks for Ag/CeO_2_ NC, particularly at 2θ = 28.62^ο^, 33.18^ο^, 47.53^ο^, 56.39^ο^, and 59.21^ο^, are in excellent agreement with JCPDS 34–0394 and findings reported by Negi et al.^32^. and Chaudhari et al.^35^.The absence of sharp Ag silver peaks conforms to other findings indicating low concentrations or evenly dispersed Ag nanoparticles, where Ag peaks can be embedded in the CeO_2_ matrix. Similarly, Ag/Bi_2_O_3_ NC XRD pattern showed strong and sharp peaks for Bi_2_O_3_ planes such as (120), (101), (200), and (311) extremely similar to Li et al.^36^., where they proved the presence of Bi_2_O_3_ in monoclinic or tetragonal phases. The presence of sharp and high-intensity peaks, lack of impurity phases, and consistency with standard diffraction patterns all validate high crystalline quality and successful embedding of silver in CeO_2_ and Bi_2_O_3_ nanomatrices, which is evidence of successful synthesis of the desired nanocomposites.

For evaluating the preliminary physicochemical stability, the synthesized Ag/CeO_2_ and Ag/Bi_2_O_3_ nanocomposites were kept in airtight containers at room temperature for a maximum period of one month. From the results, it was seen that the nanocomposites showed stability in maintaining their structural integrity, color, and dispersion properties, and there was no significant change in the position and intensity of the XRD peaks. The long-term stability is to be studied in future work.

### Phytochemical Profile

**Fig. 7:**
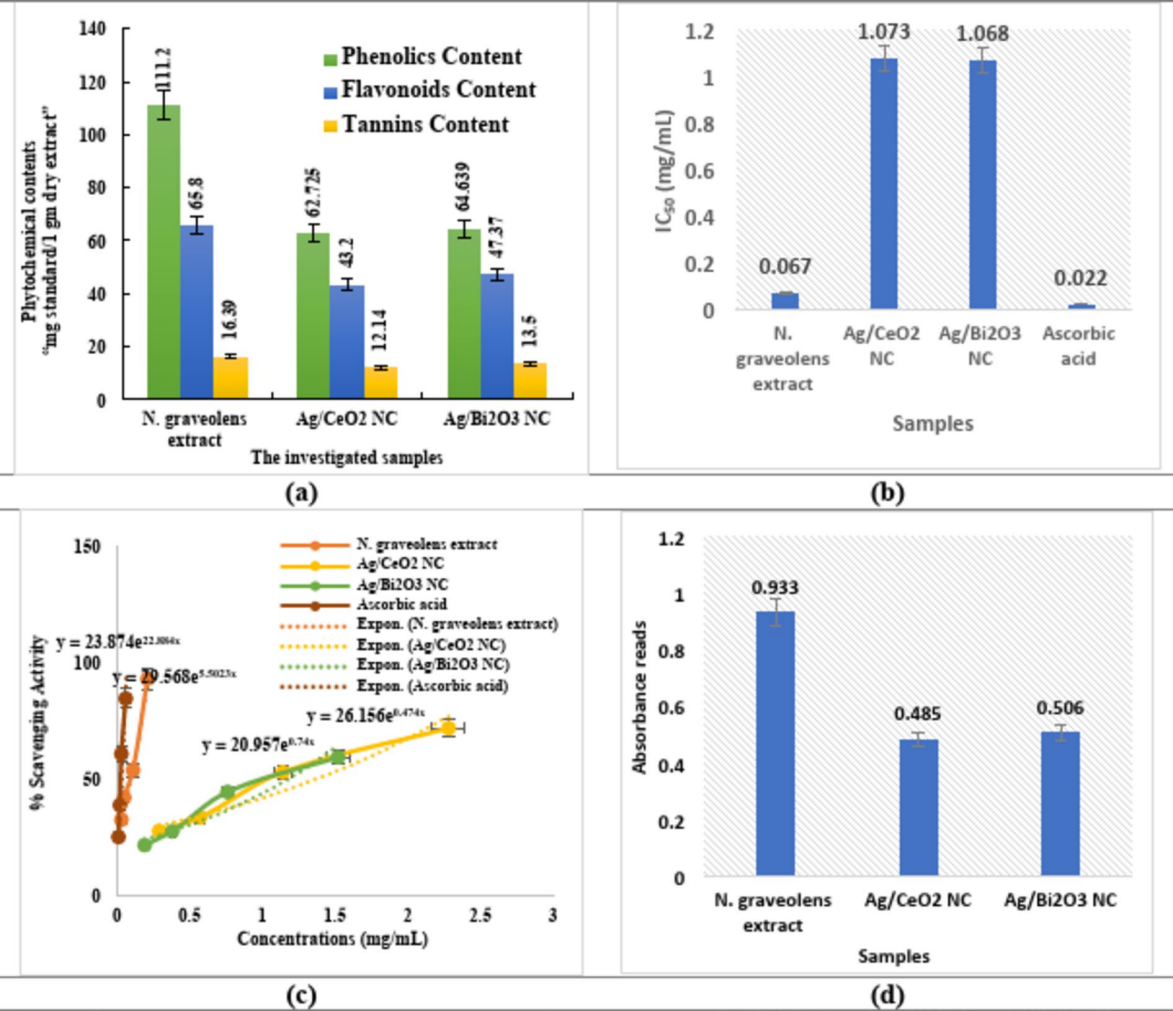
Phytochemical analysis and antioxidant results by DPPH assay. (**a**) A comparison of the phytochemical contents of the investigated samples. (**b**) A comparison of the IC_50_ values. (**c**) A plotted relationships of the concentration versus % scavenging activity. (**d**) Antioxidant results by ferric reducing power assay.

The composition of the phytochemicals in *N. graveolens* extract and the derived nanocomposites (Ag/CeO_2_ NC and Ag/Bi_2_O_3_ NC) was established as the total phenolics, flavonoids, and tannins content, as shown in Fig. 7a **& Table S6**. The crude extract of *N. graveolens* had the highest concentration of the three phytoconstituents, namely phenolics 111.2 ± 0.27 mg GAE/g, flavonoids 65.8 ± 0.16 mg CE/g, and tannins 16.39 ± 0.09 mg TAE/g. After nanocomposite formation, phytochemical content declined notably. An example of such decreased phenolic content of Ag/CeO_2_ NC was reduced to 62.725 ± 0.81 community GAE/g, flavonoids to 43.20 ± 0.34 mg CE/g, and tannins to 12.14 ± 0.34 mg TAE / g. In the same way, Ag/Bi_2_O_3_ NC showed slightly higher phytochemical values (phenolics: 64.639 ± 0.95, flavonoids: 47.37 ± 1.06, tannins: 13.5 ± 0.81) as compared to Ag/CeO_2_.

Comparable phytochemical content of both *A. graveolens* callus and parent plant material from the Algerian Sahara was also evidenced by Belhadi et al.^37^., including flavonoid and phenolic acid richness. Likewise, Mecheri et al.^38^. confirmed the presence of polyphenolic and flavonoid constituents and assigned these constituents to the plant’s high antioxidant and protective activities against oxidative stress induced by doxorubicin in vivo. Additionally, Gad et al.^24^ established that the ethanol extract of *Nauplius* (synonym of *Asteriscus*) *graveolens* exhibited vigorous anti-colorectal cancer and antimicrobial activities, which they assigned to its high polyphenols and flavonoid content.

### Antioxidant activity

#### DPPH assay

The antioxidant activity of *N. graveolens* extract, Ag/CeO_2_, Ag/Bi_2_O_3_ NCs, and ascorbic acid was investigated by DPPH radical scavenging assay, with the results summarized in Figs. 7b, c & S3 **& Table S7**. Ascorbic acid, as the positive control, demonstrated the highest antioxidant activity of IC_50_ = 0.022 mg/mL, confirming its strong free radical scavenging ability. Among all the samples tested, *N. graveolens* extract manifested considerable antioxidant capacity, as manifested by an IC_50_ value of 0.067 mg/mL, comparable to performance better than that of both nanocomposites. Ag/CeO_2_ NC possessed moderate scavenging activity (IC_50_ = 1.073 mg/mL), while Ag/Bi_2_O_3_ NC presented a slightly increased scavenging effect (IC_50_ = 1.068 mg/mL).

The lowering of the antioxidant capacity of *N. graveolens* extract after nanoparticle synthesis has been noted and can be attributed to the participation of its phytochemical compounds in bioreduction. Bioactive molecules (flavonoids, phenolics, terpenoids, and other reducing compounds of the plant extract) donate electrons in the reduction of the metal ions (Ag^+^, Ce^4+^, Bi^3+^) to their nanoparticle form during the production of Ag/CeO_2_ and Ag/Bi_2_O_3_ nanocomposites. Thus, some of the compounds containing antioxidants are lost or structurally altered during synthesis, resulting in a marked decline in the total antioxidant activity of the final nanocomposites as compared to the crude extract.

In this work, the DPPH free radical scavenging activity of the synthesized nanocomposites revealed moderate antioxidant activity owing to the phytochemicals involved in the synthesis. This is consistent with Ramdane et al.^39^., who observed significant antioxidant activity in *Asteriscus graveolens* extracts prior to any chemical alteration, where this is consistent with richness in natural phytoconstituents. Similarly, Aljeldah^11^ had exhibited significant antioxidant activity in essential oils of the same plant species. In another route, Mousa et al.^40^. had exhibited a significant loss of antioxidant activity of *Sargassum latifolium* on its use in the preparation of Ag/Bi_2_O_3_-curdlan nanocomposites, further substantiating the fact that phytochemical content is utilized actively in nanoparticle synthesis. Additionally, Mohamed et al.^41^. have documented moderate DPPH scavenging activity (~ 50%) of monoclinic α-Bi_2_O_3_ nanorods, indicating that naked metal oxides in themselves can be antioxidant in character, but generally less than their plant-extract precursors.

#### Ferric reducing power (FRAP) assay

FRAP assay was employed to measure the electron-donating capacity of the plant extract and its nanocomposites, indicating their potential antioxidant activity (Fig. 9d **& Table S8**). The absorbance values at 700 nm are consistent with the reducing power of each sample, with increased absorbance indicating better ferric ion (Fe^3+^) reduction activity. *N. graveolens* extract exhibited the highest reducing capacity, at 0.933 absorbance and 6.78 mg/mL concentration. The high reducing power is attributed to the presence of polyphenols, flavonoids, and other phytochemicals that can donate electrons towards the neutralization of free radicals or the reduction of ferric ions. Alternatively, the synthesized nanocomposites showed lower values for absorbance at the same concentration, and Ag/CeO_2_ and Ag/Bi_2_O_3_ NCs showed values of 0.485 and 0.506, respectively. Nevertheless, the fact that both the nanocomposites retain measurable reducing power indicates that there are some phytochemicals still attached to the nanomaterials, allegedly serving as stabilizing or capping agents.

These findings are in agreement with the results of the DPPH radical scavenging assay. In both assays, the *N. graveolens* extract exhibited stronger antioxidant activity than the synthesized Ag/CeO_2_ and Ag/Bi_2_O_3_ NCs. The same trend once again proves the inference that the crude extract contains a higher number of redox-active compounds, while the nanocomposites exhibit partial antioxidant activity because of the incorporation of these phytochemicals into nanoparticle formation.

**<** Fig. 9**>**.

### Antibacterial activity

**Fig. 8:**
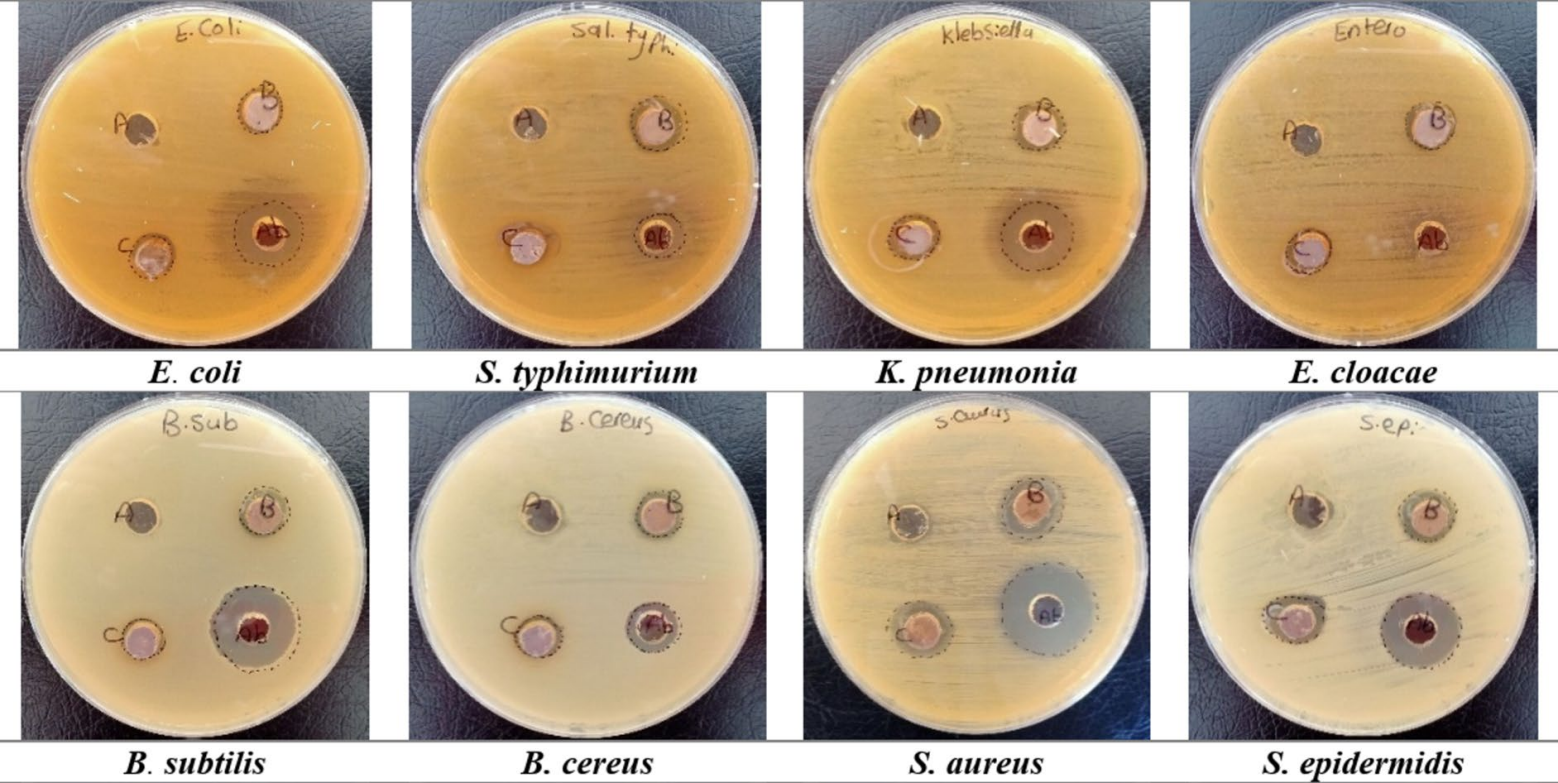
The Petri dish images described the antibacterial activity of the tested samples, and antibiotic against numerous pathogenic bacteria. (A) Referred to *A. graveolens* extract. (B) Described to Ag/CeO_2_ NC. (C) described to Ag/Bi_2_O_3_ NC, and (Ab) described to antibiotic “Azithromycin”.

The antibacterial activity of plant extract and nanocomposites was evaluated against various pathogenic bacteria (**Table S9 &** Figs. 8 & S4). Ag-CeO_2_ NC showed outstanding antibacterial capabilities since they inhibited the growth of *S. aureus*, *S. epidermidis*, and *S. typhimurium* species with zone diameters of 17.0 ± 1.83, 14.0 ± 1.77, and 14.0 ± 1.43 mm, respectively. The antibacterial properties of Ag/Bi_2_O_3_ NC were privileged against *S. aureus* and *S. epidermidis* with inhibition zones at 18.0 ± 1.83 and 15.0 ± 1.16 mm, respectively. Ag/Bi_2_O_3_ NC also verified superior resistance to *B. subtilis* with an inhibition zone reaching 11.0 ± 1.71 mm. However, the influence of Ag/Bi_2_O_3_ NC on *E. cloacae* was measured at 12.0 ± 1.38 mm, indicating a moderate resistance compared to other bacterial species.

The mechanisms behind the kinetic inhibition of bacterial strains by silver ions include significant cellular damage from silver-ion toxicity and interactions of silver nanoparticles with cells and bacterial proteins that disturb membrane functions and enzyme activity, resulting in bacterial death^42^. Bimetallic nanoparticles exhibit powerful antibacterial behavior through the combination of multiple properties that generate extra reactive oxygen species impacting on the growth of bacterial cells.

These data indicated that Ag/CeO_2_ and Ag/Bi_2_O_3_ nanocomposites are very good antibacterial agents, especially against Gram-positive bacteria, as has been shown with Ag-based nanomaterials. The superior performance against *S. aureus* and *S. epidermidis* implies that the thick peptidoglycan would bind well, and the poorer performance against Gram-negative bacteria like *E. coli* may be owing to the protective outer membrane layer^43^. Notably, Ag/CeO_2_ NC exhibited an identical or even better activity against *S. typhimurium* than azithromycin, and thus, the potential role of such nano-materials in fighting drug-resistant bacteria is highlighted.

However, it should be noted here that the results derived from the zone of inhibition assays are qualitative or semi-quantitative in nature. Such results are not necessarily indicative of the minimum inhibitory concentration (MIC) or the minimum bactericidal concentration (MBC) of the nanocomposites. This is because the diffusion of nanoparticles in agar depends on nanoparticle size, surface charge, and diffusion coefficient. Therefore, it should be noted here that the comparison of the nanocomposites with conventional antibiotics such as azithromycin may not be completely accurate. This is because the rate of diffusion of conventional antibiotics will necessarily be higher in comparison to nanoparticles due to the relatively smaller molecular weight. Nevertheless, it should be noted here that the results derived from the zone of inhibition assays are quite sufficient to ascertain the inherent antibacterial properties of the nanocomposites synthesized in this study. The combination of silver with CeO_2_ or Bi_2_O_3_ seems to have a synergetic effect, which may be the result of higher ROS production and cell membrane damage^44^. The findings are in agreement with other studies on silver nanocomposites^6^ and confirm that the hypothesis in support of the possibility of multimodal antimicrobial effects due to the hybrid and bimetallic nanostructures is also reliable.

### Insecticidal activity

**Fig. 9:**
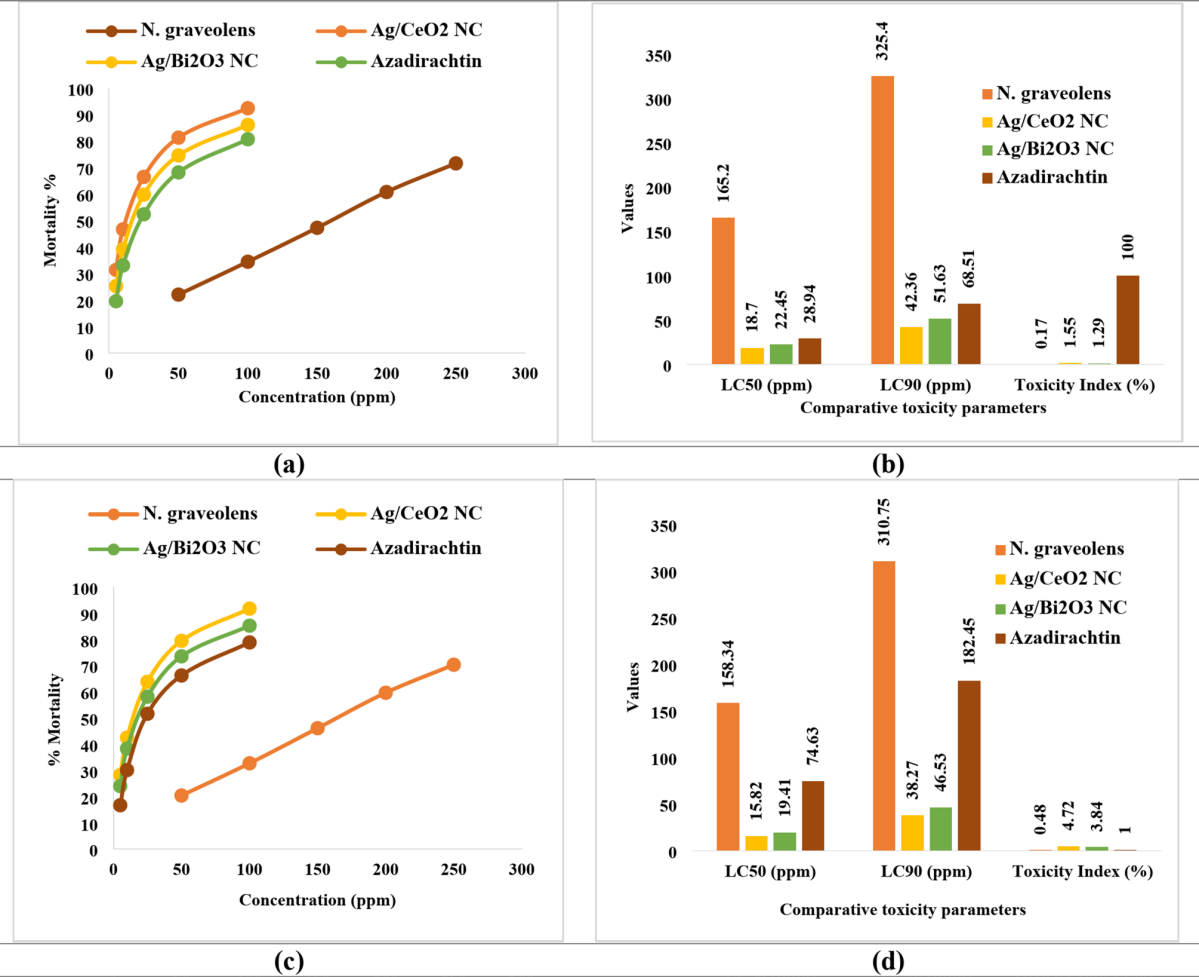
(**a**) Percentage mortality of *Aphis craccivora* after one day of exposure to the investigated samples and the standard at different concentrations (ppm) under laboratory conditions. Statistical analysis was performed using LSD at a 0.05 significance level, with values of LSD_0.05_ = 5.4 (*P* < 0.001***, *N. graveolens*), 4.7 (*P* = 0.001***, Ag/CeO_2_ NC), 5.1 (*P* < 0.001***, Ag/Bi_2_O_3_ NC), and 4.9 (*P* = 0.002**, azadirachtin). (**b**) Comparative toxicity parameters (LC_50_, LC_90_, and Toxicity Index %) of *N. graveolens* extract, Ag/CeO_2_ NC, Ag/Bi_2_O_3_ NC, and azadirachtin against *Aphis craccivora* under laboratory conditions. (**c**) Percentage mortality of *Aphis craccivora* after one day of exposure to the investigated samples and the standard at different concentrations (ppm) under laboratory conditions. Statistical analysis was performed using LSD at a 0.05 significance level, with values of LSD_0.05_ = 5.32 (P = < 0.001***, *N. graveolens*), 4.95 (P = < 0.001***, Ag/CeO_2_ NC), 5.13 (P = < 0.001***, Ag/Bi_2_O_3_ NC), and 4.71 (*P* = 0.002*, azadirachtin). (**d**) Comparative toxicity parameters (LC_50_, LC_90_, and Toxicity Index %) of*N. graveolens* extract, Ag/CeO_2_ NC, Ag/Bi_2_O_3_ NC, and azadirachtin against *Brevicoryne brassicae* under laboratory conditions. Azadirachtin was used as the reference standard (Toxicity Index = 100%).

Bioassay against *Aphis craccivora* indicated insecticidal properties in such a way that the plant extract (*N. graveolens*) and the green-synthesized nanocomposites demonstrated dose-dependent mortality within an extended concentration range of 5-250 ppm after 24 h of treatment (**Table S10 &** Figs. 9a, b). Among treatments, *N. graveolens* extract had moderate insecticidal activity such that mortality increased from 22.1 ± 2.4% with 50 ppm to 71.6 ± 3.8% with 250 ppm. The calculated LC_50_ and LC_90_ values were 165.2 ppm and 325.4 ppm, respectively, indicating relatively lower potency (Toxicity Index = 0.17) compared to the nanocomposites. Both the nanocomposite formulations, on the contrary, exhibited significantly enhanced insecticidal activity at significantly reduced concentrations. Ag/CeO_2_ NC exhibited the highest bioactivity with 92.4 ± 2.1% mortality at 100 ppm, and LC_50_ and LC_90_ values of 18.7 ppm and 42.36 ppm, respectively. The expanded concentration range allowed more accurate dose–response curves, improving the reliability of LC_50_, LC_90_, and toxicity index comparisons. Interestingly, both nanocomposites had higher efficacy compared to the commercial reference azadirachtin (LC_50_ = 28.94 ppm; LC_90_ = 68.51 ppm; Toxicity Index = 100%), proving their value as green substitutes for pest control. In general, the present findings confirm that the nanocomposites represent a new and sustainable approach to insect pest control.

The bioassays against *Brevicoryne brassicae* demonstrated dose-dependent mortality for all the test treatments (**Table S11 &** Figs. 9c, d). The *N. graveolens* extract was moderately toxic, such that mortality increased from 20.5 ± 2.1% at 50 ppm to 70.4 ± 3.6% at 250 ppm. The LC_50_ and LC_90_ values were calculated as 158.34 ppm and 310.75 ppm, respectively, with a toxicity index of only 0.48, indicating poor efficacy compared to the nanocomposites and azadirachtin. On the other hand, nanocomposites were more effective at significantly lower doses. Ag/CeO_2_ NC was the most effective, causing 91.8 ± 2.3% mortality at 100 ppm and had LC_50_ and LC_90_ values of 15.82 ppm and 38.27 ppm, respectively, with the steepest dose-response slope (3.08 ± 0.25). Ag/Bi_2_O_3_ NC also showed excellent insecticidal activity with LC_50_ = 19.41 ppm and LC_90_ = 46.53 ppm, with 3.84 as the toxicity index. The broader dose-response assessment strengthens the reliability of these comparisons and confirms the superior efficacy of nanocomposites over both the plant extract and commercial azadirachtin (LC_50_ = 74.63 ppm; LC_90_ = 182.45 ppm; Toxicity Index = 1.00).

Enhanced insecticidal activity of green-synthesized nanocomposites over *N. graveolens* extract is attributed to their multimodal modes of action. Synergistic integration of silver with transition metal oxides such as CeO_2_ or Bi_2_O_3_ promotes excessive ROS production, which generates oxidative stress, lipid peroxidation, protein denaturation, and DNA fragmentation in insect cells^45–47^. Moreover, silver ions create a complex upon binding with membrane protein sulfhydryl groups, destabilizing structural integrity, permeabilizing the membrane, and ultimately causing cell lysis^46,48^. These interactions are often associated with inhibition of enzymatic repair processes, further accelerating cellular degradation^45,46^. The nanoscale dimension and high surface reactivity of these composites enable ready penetration through the aphid cuticle and therefore enhance systemic bioavailability at lower concentrations compared to the modest bioactivity of raw phytochemicals^45,46,49^. In addition, ROS generated from CeO_2_ and Bi_2_O_3_ nanoparticles inhibit key antioxidant defense enzymes, including catalase, superoxide dismutase, and glutathione-*S*-transferase. Silver ions, however, destabilize mitochondrial function and energy metabolism, leading to metabolic failure in the end^46,47^. Collectively, these physical barriers and biochemical disruptions render Ag/CeO_2_ and Ag/Bi_2_O_3_ NCs effective and green alternatives to crude extracts or synthetic pesticides^45–49^.

**Fig. 10:**
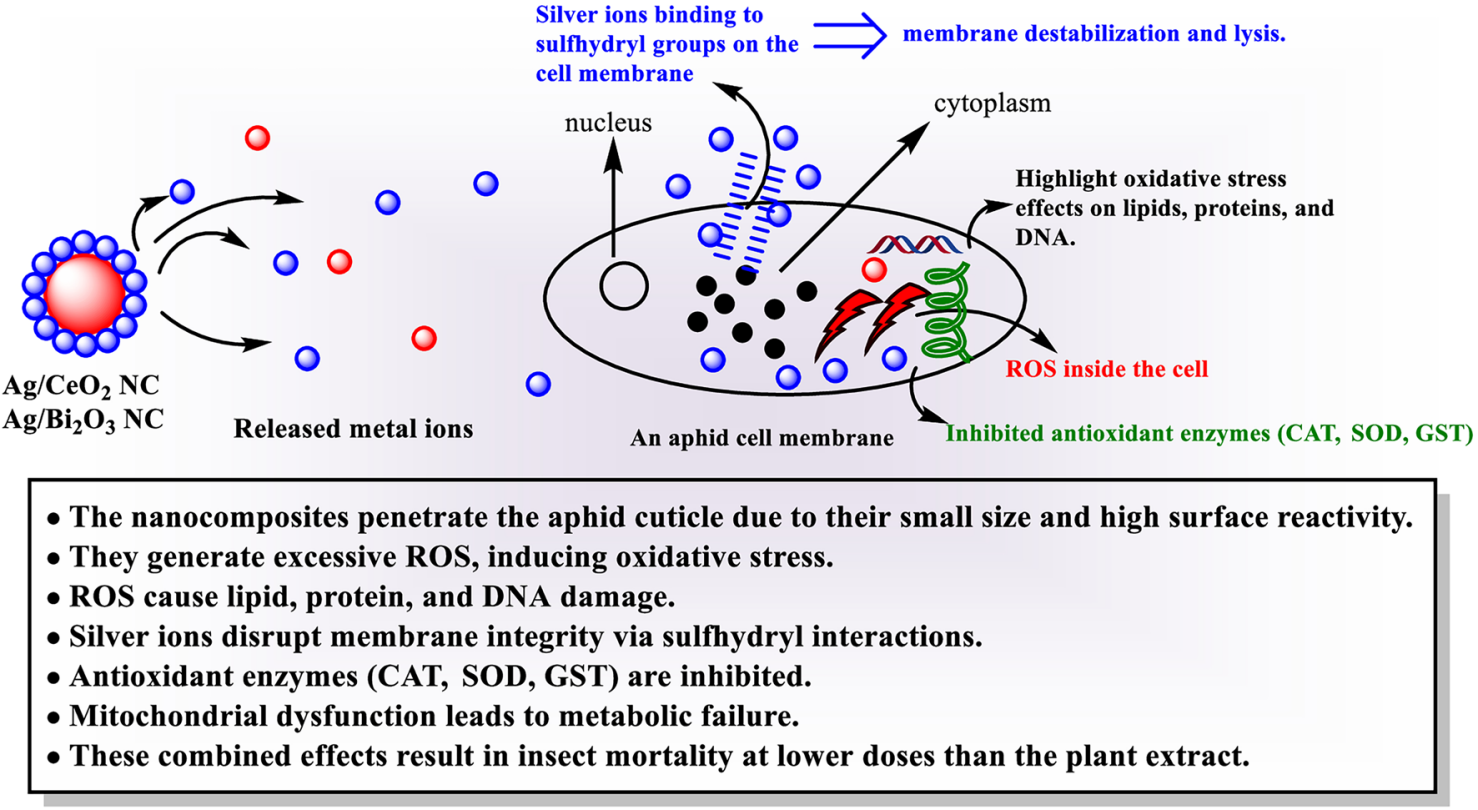
Proposed insecticidal mechanism of Ag/CeO_2_ and Ag/Bi_2_O_3_ nanocomposites against aphid pests. The mechanism by which the compound acts also shows its multi-target insecticidal activity. The insecticidal nanocomposites have ROS-mediated oxidative stress, disruption of the cellular membrane by interaction with silver and sulfhydryl groups, and inhibition of antioxidant enzyme activity. The small size and high reactivity of the compound also improve penetration and increase systemic availability compared to the plant extract.

The mechanism of the proposed nanocomposites is demonstrated in the following schematic diagram (Fig. 10). The nanocomposites’ mechanism of action against the aphid pests follows a multi-target approach: ROS generation by Ag, CeO_2_, and Bi_2_O_3_ nanoparticles generates oxidative stress that leads to lipid peroxidation, denaturation of proteins, and DNA damage; damage to the cell membrane is due to binding of the sulfhydryl groups of membrane proteins with silver ions, leading to cell lysis; enzyme inhibition is due to inhibition of key antioxidant enzymes like catalase (CAT), superoxide dismutase (SOD), and glutathione S-transferase (GST). The nanoscale of the nanocomposites and high surface activity make them easier to penetrate the cuticles and increase their availability in the system, thus making them more effective insecticides even at lower doses than those used in the plant extract.

Though the nanocomposites showed potent insecticidal activity at lower concentrations, the environmental persistence of these nanocomposites is likely to be short due to the natural biodegradability of plant-derived compounds and the tendency of nanosilver and metal oxide nanoparticles to aggregate and settle in soil and water. Future research is needed to assess the environmental fate and ecotoxicity of these nanocomposites.

## Conclusion

The current work successfully demonstrated a green route for the synthesis of Ag/CeO_2_ and Ag/Bi_2_O_3_ nanohybrids through the phytochemical-rich extract of *N. graveolens*. The detailed characterization demonstrated the successful synthesis of well-defined, crystalline nanocomposites with the silver nanoparticles well embedded in the metal oxide lattices. The plant extract and synthesized nanohybrids were observed to be highly antioxidant in activity. More importantly, the Ag/CeO_2_ and Ag/Bi_2_O_3_ nanohybrids were discovered to have good antibacterial activity, particularly against Gram-positive bacteria. It must be noted, however, that the potential therapeutic application of the nanohybrids should not be immediately concluded. Rather, the increased bioactivity of the nanohybrids may be attributed to the synergistic interaction between the silver nanoparticles and the metal oxides, which increases the efficiency of surface reactivity and biological interaction.

From the results of the insecticidal bioassay, it was revealed that the bioactivity of the prepared nanocomposites using *N. graveolens* plant extract was found to be significantly higher compared to the plant extract and azadirachtin. This reveals the potential for the synthesized nanocomposites in the development of novel methods for pest control in the future, even though their environmental safety, selectivity, and efficiency are still to be confirmed. This reveals the functional potential of the green-synthesized nanohybrids, but their use in agriculture or medicine on a large scale cannot be ascertained in the present context. Future studies could be directed towards the evaluation of the in vitro cytotoxicity using non-target mammalian cell lines, in addition to in vivo or ex vivo studies, in order to ascertain the safe dosage levels for the use of these nanohybrids.

## Supplementary Information

Below is the link to the electronic supplementary material.

Supplementary Material 1

Supplementary Material 2

## Data Availability

All applicable data are within the manuscript and obtainable from the corresponding author upon request.
