# Supplementary material for: Bioinspired Ag/CeO2 and Ag/Bi2O3 nanohybrids synthesized with Nauplius graveolens for antioxidant, antibacterial, and insecticidal applications
Source: Sci Rep. 2026 Apr 19;16:12879. doi: 10.1038/s41598-026-42713-7 (PMC13096474; doi:10.1038/s41598-026-42713-7)
Supplement: Supplementary file 1 — Supplementary Material 1 [file 41598_2026_42713_MOESM1_ESM.docx]

**Supplementary Data**

**Bioinspired Ag/CeO_2_ and Ag/Bi_2_O_3_ Nanohybrids Synthesized with *Nauplius graveolens* for Antioxidant, Antibacterial, and Insecticidal Applications**

**Section S1: Material and Methods**

**Reagents**:

Folin-Ciocalteu reagent (analytical grade, Fluka, Biochemical Inc., Bucharest, Romania), Gallic acid (≥98%, Biomedical Inc., Orange City, FL, USA), 1,1-Diphenyl-2-picrylhydrazyl (DPPH^•^) (≥97%), aluminum chloride (anhydrous, ≥99%), sodium hydroxide (pellets, ≥99%), sodium nitrite (≥99%), catechin hydrate (≥98%), vanillin (≥99%), hydrochloric acid (37%), and ascorbic acid (≥99%) were purchased from Sigma Aldrich (St. Louis, USA). Sodium Carbonate (analytical grade), and tannic acid (≥98%) were purchased from El-Nasr Pharmaceutical Chemicals, Cairo, Egypt. Silver nitrate (AgNO_3_, 99%), cerium dioxide (CeO_2_), 99%) and bismuth oxide (Bi_2_O_3_, ≥ 98%) were purchased from PIOCHEM laboratory chemicals.

**Instruments**

UV-Vis spectroscopy (Spekol 11, analytic Jena AG, Jena, Germany) was used to investigate optical properties. At the same time, SEM (Czech FEI SEM-type instrument) provided insights into morphology of the nanoparticles. To characterize nanoparticles, a high-resolution transmission electron microscope (HR-TEM) was performed on Thermo Scientific Talos F200i. X-ray diffraction (XRD) analyses were executed on a Pan Analytical Philips. Sonication was performed using an Elma Schmidbauer GmbH (Gottlieb-Daimler-Straße 17, Singen, Germany) sonicator during nanoparticle preparation. Subsequently, a Beckman Coulter Allegra X-15R centrifuge (Beckman Coulter, Inc., California, USA) was employed to isolate the purified nanoparticles.

**GC-MS Analysis**

GC-MS analysis of *N*. *graveolens* ethanol extract was performed on a Trace GC-TSQ mass spectrometer (Thermo Scientific, Austin, TX, USA) with a TG-5MS capillary column (30 m × 0.25 mm × 0.25 µm film thickness) [1]. The oven temperature was set at first to 50 °C, and subsequently to 250 °C at 5 °C/min and held for 2 minutes. This was then followed by a rise of another 30 °C/min to a final temperature of 300 °C, also held for 2 minutes. The MS transfer line and injector were held at 260 °C and 270 °C, respectively. Helium was used as the carrier gas with a set flow rate of 1 mL/min. After a 4-minute solvent hold, 1 µL of diluted sample was injected in split mode using an Autosampler AS1300. Mass spectra were recorded in electron ionization (EI) mode from m/z 50 to 500 at an ionization voltage of 70 eV, with the temperature of the ion source at 200 °C. Compound identification was achieved by comparing the obtained mass spectra against WILEY 09 and NIST 14 mass spectral libraries. For each detected peak, up to five potential compound matches were suggested, with final identifications based on the highest probability scores and consistent fragmentation patterns.

**Phytochemical Analysis**

***1. Total Tannin Contents***

Tannin content was estimated using the vanillin-hydrochloride method outlined above [2]. Vanillin-hydrochloride reagent was prepared by mixing equal volumes of 30% hydrochloric acid in methanol and 4% vanillin solution in methanol. For the assay, 5 mL of the above-prepared reagent was added to 1 mL of the sample solution. The room temperature incubation of 20 minutes was done with the mixture, and the orange color produced was read spectrophotometrically at 510 nm. The tannin content was expressed in terms of tannic acid equivalents (TAE) from a standard calibration curve made utilizing tannic acid (Y = 0.0009X, R^2^ = 0.955), and results were reported as g TAE per dry sample 1 g.

***2. Total Phenolic Content***

Total phenolic content (TPC) was assessed with the Folin-Ciocalteu assay by previous report [3]. A sample was added to a cuvette, 100 µL to 5 mL of diluted Folin-Ciocalteu reagent in a dilution ratio of 1:10 in distilled water. 4 mL of sodium carbonate solution at a concentration of 7.5% was added and well mixed. Distilled water up to 10 mL. The reaction mixture was then incubated in the dark at 40 °C for 30 minutes. The absorbance of the resulting blue-colored solution at 765 nm was then determined by a UV-Vis spectrophotometer after incubation. A calibration graph was obtained with 0-100 mg/L solutions of Gallic acid. The phenolic content of all the samples was approximated by interpolation of their absorbance on the Gallic acid standard curve (y = 0.0062x, R^2^ = 0.987) and was reported in terms of milligrams of Gallic acid equivalents per gram dry weight (mg GAE/g DW).

***3. Total Flavonoid Content***

The flavonoids of the samples were determined using the colorimetric aluminum chloride method [4]. The sample of approximately 100 μL was transferred to a cuvette, then 4 mL of distilled water and 0.3 mL of 5% sodium nitrite solution were added. The test mixture was left at room temperature for 5 minutes. Then 0.3 mL of the aluminum chloride solution (10% ethanol) was added, shaken well, and incubated for another 5 minutes. 2 mL of 1 M sodium hydroxide was then added, shaken well, and the reaction mixture was left at room temperature for 15 minutes. Distilled water was added to the reaction mixture to make it up to 10 mL. Absorbance at 510 nm of the orange solution was taken using a spectrophotometer. A calibration curve over a concentration range of 0-100 mg/L catechin was constructed. Flavonoid content was determined by interpolating the absorbance of the sample on the catechin standard curve (y = 0.0028x, R^2^ = 0.988) and expressed as milligrams of catechin equivalents per gram of dry weight (mg CE/g DW).

**Antioxidant activity**

**1. DPPH assay**

The antioxidant capacity of the tested samples was investigated following the DPPH^•^ colorimetric method using ascorbic acid as a standard [5]. The serial dilution of each sample was prepared by mixing the sample with methanol in an equivalent amount. DPPH^•^ solution was prepared in a concentration of 0.135 mM and mixed with each sample in the serial dilution with an equivalent volume. After the addition of DPPH^•^ solution, the samples were kept in dark for 30 minutes at room temperature. The absorbance of each sample was measured at 517 nm in the next step. The % DPPH^•^ remaining was calculated stratifying the subsequent equation (Eq. 1):

% DPPH^•^ remaining = [DPPH^•^]_T_/ [DPPH^•^]_T= 0_ x 100 Eq. (1)

The values of % DPPH^•^ remaining were plotted versus the sample concentration in mg /mL using an exponential curve to identify the effective concentration “IC_50_”. IC_50_ indicated the constituted amount of antioxidants needed to decrease the initial concentration of DPPH^•^ solution by 50%. The values of IC_50_ point out the inverse relationship with the antioxidant capacity of the tested sample.

**2. Ferric-reducing power assay**

The reducing power of the extract and nanocomposites was assayed by the modified method of previous studies [6]. A mixture of the tested samples (1 mL) was added to phosphate buffer (2.5 mL) (0.2 M, pH 6.6), and potassium ferrocyanide (2.5 mL, 1%). The mixture was incubated at 50 °C for 20 minutes. Trichloroacetic acid (2.5 mL, 10%) was added to the mixture. Centrifugation was accomplished at 3000 rpm for 10 min to discard the precipitate. After transferring 2.5 mL of supernatant with 2.5 mL distilled water, 0.5 mL of 0.1% FeCl_3_ solution was added to the mixture and the absorbance was determined at 700 nm. The absorbance reads were compared with that of ascorbic acid “a reference standard”.

**Bacterial species**:

Gram-negative bacteria (*E. coli* ATCC 10536, *S. typhimurium* ATCC 25566, *K. pneumoniae* ATCC 10031, and *E. cloacae* DMS 30054) and Gram-positive bacteria (*B. subtilis* DMS 1088, *B. cereus* EMCC 1080, *S. aureus* ATCC 6538, and *S. epidermidis* EMCC 1353t) were obtained from the Microbiological Resources Centre, Cairo, Egypt (Mircen), and the American Type Culture Collection (ATCC, Manassas, VA, USA).

**Section S2: Results**

**GC-MS Spectroscopy**

**Table S1**. The results of the GC/MS analysis of the volatile components extracted from *N. graveolens.*

| Entry | Compounds | Classification | Retention time (RT, min) | Molecular Weight | Molecular formula | Composition % |
| --- | --- | --- | --- | --- | --- | --- |
| 1 | (1R,5S,6R)-2,7,7-trimethylbicyclo[3.1.1]hept-2-en-6-yl acetate | Hydrocarbon | 11.05 | 194.27 | C_12_H_18_O_2_ | 2.0 |
| 2 | 6-allyl-4-methoxybenzo[d][1,3]-dioxole | Hydrocarbon | 17.22 | 192.21 | C_11_H_12_O_3_ | 3.84 |
| 3 | 7-epi-cis-sesquisabinene hydrate  “*(1S,2R,5R)-2-methyl-5-[(R)-6-methylhept-5-en-2-yl]bicyclo[3.1.0]hexan-2-ol*” | Sesquiterpene | 20.29 | 222.37 | C_15_H_26_O | 0.53 |
| 4 | oleic acid | Fatty acid | 20.93 | 282.47 | C_18_H_34_O_2_ | 4.01 |
| 5 | Shyobunol “*(1R,2S,3S,6S)-6-isopropyl-3-methyl-2-(prop-1-en-2-yl)-3-vinylcyclohexan-1-ol*” | Sesquiterpene | 21.38 | 222.37 | C_15_H_26_O | 1.52 |
| 6 | Corymbolone “(4S,4aR,6R,8aR)-4a-hydroxy-4,8a-dimethyl-6-prop-1-en-2-yl-3,4,5,6,7,8-hexahydro-2H-naphthalen-1-one” | Sesquiterpene | 22.64 | 236.36 | C_15_H_24_O_2_ | 20.11 |
| 7 | (E)-5,6,6-trimethyl-5-(3-oxobut-1-en-1-yl)-1-oxaspiro[2.5]octan-4-one | Hydrocarbon | 23.20 | 236.31 | C_14_H_20_O_3_ | 0.66 |
| 8 | 10αH-Ambros-11(13)-en-12-oic acid, 3α,4α,6β-trihydroxy-, γ-lactone | Sesquiterpene | 24.01 | 266.0 | C_15_H_22_O_4_ | 0.61 |
| 9 | methyl (6E,9E,12E,15E)-docosa-6,9,12,15-tetraenoate | Lipid | 24.20 | 346.56 | C_23_H_38_O_2_ | 1.0 |
| 10 | Spirafolide | Sesquiterpene | 24.49 | 276.38 | C_17_H_24_O_3_ | 1.21 |
| 11 | methyl 14-methylpentadecanoate | Lipid | 26.41 | 270.46 | C_17_H_34_O_2_ | 0.85 |
| 12 | α-Elemenone “*(4R,5S)-5-methyl-4-(prop-1-en-2-yl)-2-(propan-2-ylidene)-5-vinylcyclohexan-1-one*” | Sesquiterpene | 26.87 | 218.34 | C_15_H_22_O | 7.90 |
| 13 | palmitic acid | Fatty acid | 27.42 | 256.43 | C_16_H_32_O_2_ | 6.76 |
| 14 | (E)-3-methyl-4-(2,6,6-trimethylcyclohex-2-en-1-yl)but-3-en-2-one | Hydrocarbon | 27.88 | 206.33 | C_14_H_22_O | 3.61 |
| 15 | (2E,3Z)-2-ethylidene-6-methylhepta-3,5-dienal | Monoterpene | 28.13 | 150.22 | C_10_H_14_O | 4.87 |
| 16 | methyl (2E,4E)-hexadeca-2,4-dienoate | Lipid | 29.17 | 266.43 | C_17_H_30_O_2_ | 1.21 |
| 17 | methyl (7E,10E)-octadeca-7,10-dienoate | Lipid | 29.44 | 294.48 | C_19_H_34_O_2_ | 1.33 |
| 18 | methyl (E)-octadec-10-enoate | Lipid | 29.62 | 296.50 | C_19_H_36_O_2_ | 1.25 |
| 19 | (9Z,12Z)-octadeca-9,12-dienoic acid | Fatty acid | 30.57 | 280.45 | C_18_H_32_O_2_ | 8.19 |
| 20 | (E)-octadec-13-enoic acid | Fatty acid | 30.70 | 282.47 | C_18_H_34_O_2_ | 11.53 |
| 21 | oxiran-2-ylmethyl oleate | Lipid | 32.87 | 338.53 | C_21_H_38_O_3_ | 0.80 |
| 22 | methyl (7E,10E,13E)-icosa-7,10,13-trienoate | Lipid | 35.56 | 320.52 | C_21_H_36_O_2_ | 2.39 |
| 23 | Linolein, 2-mono- “*1,3-dihydroxypropan-2-yl (9Z,12Z)-octadeca-9,12-dienoate*” | Lipid | 35.69 | 354.53 | C_21_H_38_O_4_ | 2.85 |
| 24 | 2,3-dihydroxypropyl palmitate | Lipid | 36.21 | 330.51 | C_19_H_38_O_4_ | 1.91 |
| 25 | 1,3-dihydroxypropan-2-yl oleate | Lipid | 38.90 | 356.55 | C_21_H_40_O_4_ | 7.21 |
| 26 | Stigmasterol | Steroid | 44.98 | 412.70 | C_29_H_48_O | 1.84 |

**Figure S1**. A comparison between the different categories of volatile components isolated from N. graveolens.

**Characterization of Nanocomposites**

**FTIR Spectroscopy**

**Table S2**: The results of the FTIR spectra of *N. graveolens* extract and its derived nanocomposites (Ag/CeO_2_ NC and Ag/Bi_2_O_3_ NC):

| **Wavenumber (cm^-1^)** | **Functional Group** | **Interpretation** | ***N. graveolens* Extract** | **Ag/CeO_2_ NC** | **Ag/Bi_2_O_3_ NC** |
| --- | --- | --- | --- | --- | --- |
| 3293 | O-H Stretch | Hydroxyl group (phenolic, alcohols) | ✓ |  | ✓ |
| 2972 | C-H Stretch | Aliphatic C-H stretch (alkyl groups) | ✓ |  |  |
| 2921 | C-H Stretch | Aliphatic C-H stretch (alkyl groups) | ✓ |  |  |
| 2850 | C-H Stretch | Aliphatic C-H stretch (alkyl groups) | ✓ |  |  |
| 1733 | C=O Stretch | Carbonyl group (esters, aldehydes, or acids) | ✓ | ✓ | ✓ |
| 1631 | C=C Stretch (Aromatic) | Aromatic ring stretch (phenolic compounds) | ✓ |  |  |
| 1598 | C=C Stretch (Aromatic) | Aromatic ring stretch (phenolic compounds) | ✓ | ✓ | ✓ |
| 1515 | C=C Stretch (Aromatic) | Aromatic ring stretch (phenolic compounds) | ✓ |  |  |
| 1457 | C-H Bending | C-H bending (alkyl groups) | ✓ |  |  |
| 1374 | C-H Bending | C-H bending (alkyl groups) | ✓ |  |  |
| 1273 | C-O Stretch | C-O stretching (alcohols, phenols, esters) | ✓ |  |  |
| 1082 | C-O Stretch | C-O stretching (alcohols, ethers) | ✓ | ✓ | ✓ |
| 1046 | C-O Stretch | C-O stretching (alcohols, ethers) | ✓ |  |  |
| 983 | C-H Bending | C-H bending (alkyl groups) | ✓ |  |  |
| 928 | C-H Bending | C-H bending (alkyl groups) | ✓ |  |  |
| 879 | C-H Bending | C-H bending (alkyl groups) | ✓ |  |  |
| 816 | C-H Bending | C-H bending (alkyl groups) | ✓ | ✓ | ✓ |
| 777 | C-H Bending | C-H bending (alkyl groups) | ✓ |  |  |
| 716 | C-H Bending | C-H bending (alkyl groups) | ✓ |  |  |
| 480 | M-O Stretch | Metal-oxide (CeO_2_ or Bi_2_O_3_) |  | ✓ | ✓ |
| 450 | M-O Stretch | Metal-oxide (CeO_2_ or Bi_2_O_3_) |  | ✓ | ✓ |
| 418 | M-O Stretch | Metal-oxide (CeO_2_ or Bi_2_O_3_) |  |  | ✓ |
| 391 | M-O Stretch | Metal-oxide (CeO_2_ or Bi_2_O_3_) |  |  | ✓ |
| 1773 | C=O Stretch | Carbonyl group (esters, aldehydes, or acids) |  | ✓ | ✓ |
| 1751 | C=O Stretch | Carbonyl group (esters, aldehydes, or acids) |  | ✓ |  |
| 1351 | C-H Bending | C-H bending (alkyl groups) |  | ✓ |  |
| 1306 | C-O Stretch | C-O stretching (alcohols, ethers) |  | ✓ |  |
| 1070 | C-O Stretch | C-O stretching (alcohols, ethers) |  | ✓ |  |
| 1036 | C-O Stretch | C-O stretching (alcohols, ethers) |  | ✓ |  |
| 824 | C-H Bending | C-H bending (alkyl groups) |  | ✓ |  |
| 795 | C-H Bending | C-H bending (alkyl groups) |  | ✓ |  |
| 729 | C-H Bending | C-H bending (alkyl groups) |  | ✓ |  |
| 501 | M-O Stretch | Metal-oxide (CeO_2_ or Bi_2_O_3_) |  | ✓ |  |
| 473 | M-O Stretch | Metal-oxide (CeO_2_ or Bi_2_O_3_) |  | ✓ |  |
| 3048 | C-H Stretch | Aliphatic C-H stretch (alkyl groups) |  |  | ✓ |
| 1577 | C=C Stretch (Aromatic) | Aromatic ring stretch (phenolic compounds) |  |  | ✓ |
| 1456 | C-H Bending | C-H bending (alkyl groups) |  |  | ✓ |
| 1418 | C-H Bending | C-H bending (alkyl groups) |  |  | ✓ |
| 1380 | C-H Bending | C-H bending (alkyl groups) |  |  | ✓ |
| 1287 | C-O Stretch | C-O stretching (alcohols, ethers) |  |  | ✓ |
| 809 | C-H Bending | C-H bending (alkyl groups) |  |  | ✓ |
| 721 | C-H Bending | C-H bending (alkyl groups) |  |  | ✓ |
| 606 | M-O Stretch | Metal-oxide (Bi_2_O_3_) |  |  | ✓ |
| 497 | M-O Stretch | Metal-oxide (Bi_2_O_3_) |  |  | ✓ |

**UV-Visible Spectroscopy**

**Table S3**: The results of UV-visible spectroscopy of the plant extract and nanocomposites.

| Samples | Absorbance | Wavelength (nm) |
| --- | --- | --- |
| *N. graveolens* extract | 398.0 | 0.521 |
| Ag/CeO_2_ NC | 385.0 | 0.402 |
| Ag/Bi_2_O_3_ NC | 389.0 | 0.379 |
| Ag/Bi_2_O_3_ NC | 519.0 | 0.430 |

**Zeta Potential Analysis**

| 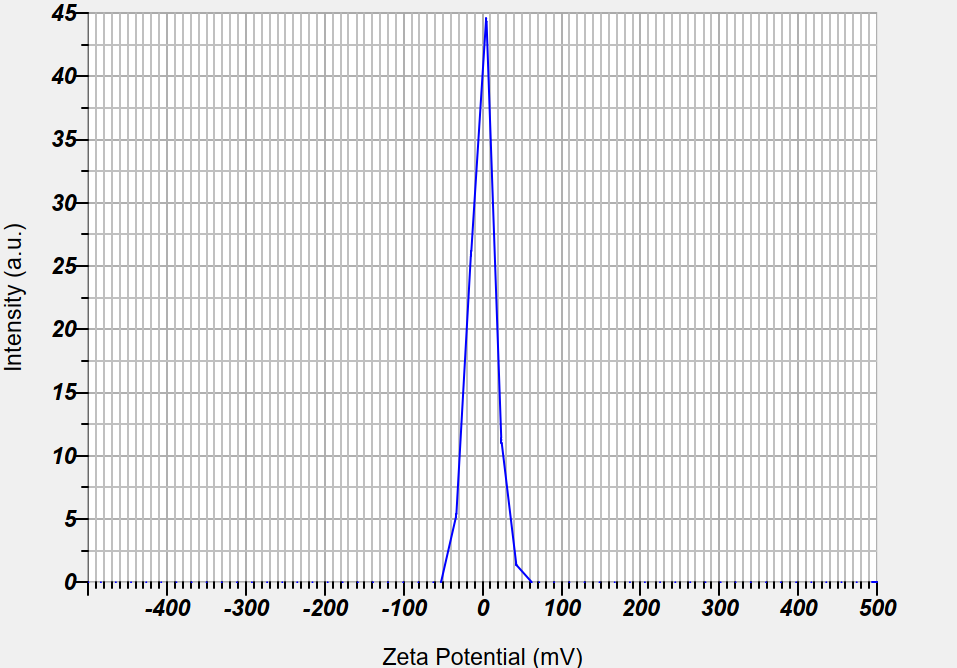 | 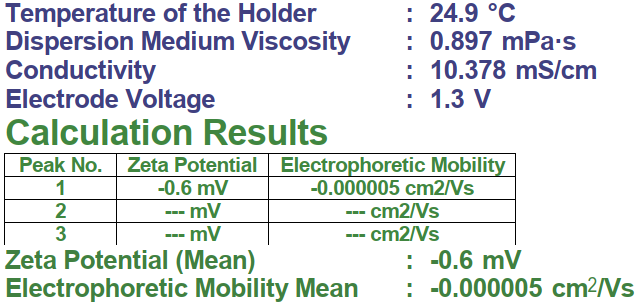 |
| --- | --- |
| (A) |  |
| 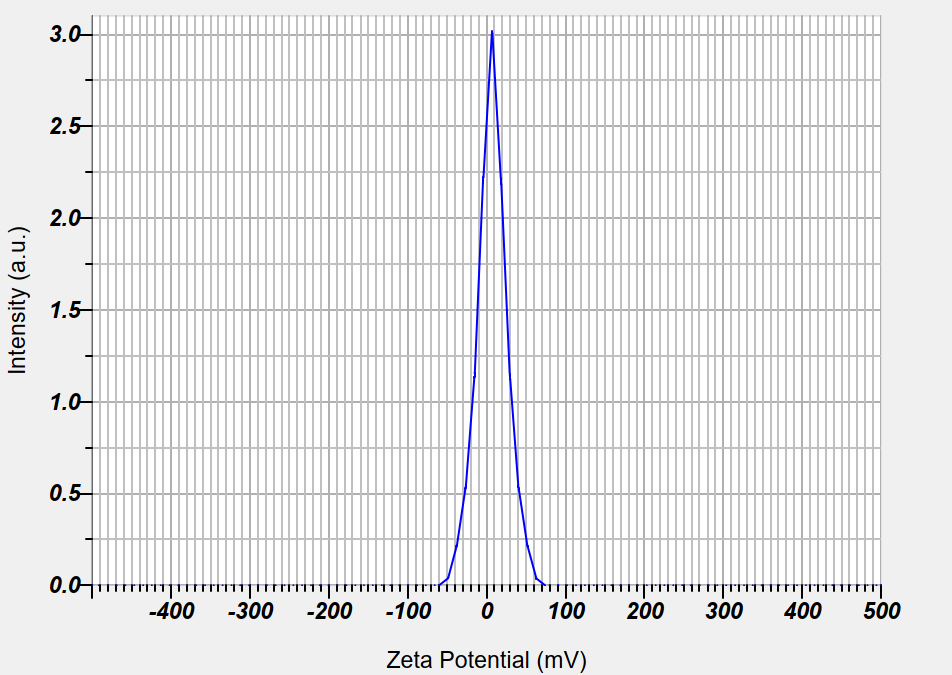 | 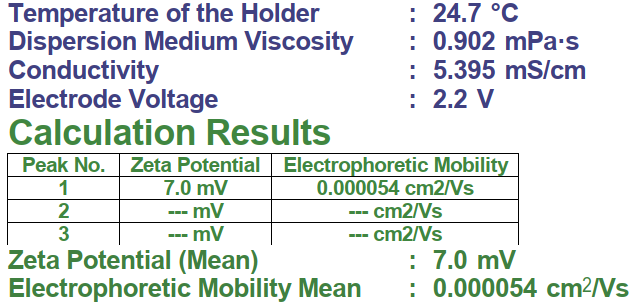 |
| (B) |  |

**Figure S2**. Zeta potential analyses of Ag/CeO_2_ NC (A), and Ag/Bi_2_O_3_ NC (B).

**X-Ray Diffraction (XRD)**

**Table S4**. Peak List identified from XRD analysis of Ag/CeO_2_ NC.

| Pos. [°2Th.] | Height [cts] | FWHM [°2Th.] | d-spacing [Å] | Rel. Int. [%] | Tip width [°2Th.] |
| --- | --- | --- | --- | --- | --- |
| 28.6204 | 249.88 | 0.0984 | 3.11903 | 100.00 | 0.1181 |
| 33.1871 | 71.20 | 0.1574 | 2.69954 | 28.49 | 0.1889 |
| 47.5360 | 92.92 | 0.2755 | 1.91283 | 37.19 | 0.3306 |
| 56.3943 | 85.01 | 0.1968 | 1.63159 | 34.02 | 0.2362 |
| 59.2163 | 13.04 | 0.3149 | 1.56039 | 5.22 | 0.3779 |
| 62.7069 | 2.29 | 0.3149 | 1.48167 | 0.92 | 0.3779 |
| 69.5320 | 12.68 | 0.3149 | 1.35198 | 5.07 | 0.3779 |
| 76.7955 | 23.01 | 0.4723 | 1.24121 | 9.21 | 0.5668 |
| 79.1184 | 23.08 | 0.1181 | 1.21050 | 9.24 | 0.1417 |
| 88.4676 | 18.78 | 0.3840 | 1.10423 | 7.52 | 0.4608 |

**Table S5**. Peak List identified from XRD analysis of Ag/Bi_2_O_3_ NC

| Pos. [°2Th.] | Height [cts] | FWHM [°2Th.] | d-spacing [Å] | Rel. Int. [%] | Tip width [°2Th.] |
| --- | --- | --- | --- | --- | --- |
| 12.9617 | 14.97 | 0.2755 | 6.83026 | 2.80 | 0.3306 |
| 23.9650 | 24.63 | 0.1574 | 3.71333 | 4.60 | 0.1889 |
| 24.6379 | 21.02 | 0.1181 | 3.61343 | 3.93 | 0.1417 |
| 25.8190 | 22.69 | 0.1181 | 3.45074 | 4.24 | 0.1417 |
| 27.0052 | 73.18 | 0.1378 | 3.30180 | 13.68 | 0.1653 |
| 27.4492 | 534.99 | 0.1181 | 3.24939 | 100.00 | 0.1417 |
| 28.0774 | 37.24 | 0.1181 | 3.17811 | 6.96 | 0.1417 |
| 30.3468 | 66.65 | 0.1968 | 2.94542 | 12.46 | 0.2362 |
| 33.0904 | 89.42 | 0.0590 | 2.70721 | 16.71 | 0.0708 |
| 33.3153 | 145.21 | 0.0590 | 2.68945 | 27.14 | 0.0708 |
| 35.0977 | 42.93 | 0.0590 | 2.55685 | 8.02 | 0.0708 |
| 35.4909 | 31.19 | 0.1574 | 2.52942 | 5.83 | 0.1889 |
| 37.0522 | 14.51 | 0.1574 | 2.42634 | 2.71 | 0.1889 |
| 37.6604 | 40.82 | 0.0590 | 2.38854 | 7.63 | 0.0708 |
| 40.1415 | 7.78 | 0.2362 | 2.24645 | 1.45 | 0.2834 |
| 41.5337 | 21.40 | 0.1181 | 2.17431 | 4.00 | 0.1417 |
| 42.4921 | 17.95 | 0.2362 | 2.12747 | 3.36 | 0.2834 |
| 46.3788 | 114.37 | 0.0590 | 1.95782 | 21.38 | 0.0708 |
| 47.6584 | 11.93 | 0.2362 | 1.90820 | 2.23 | 0.2834 |
| 48.4651 | 50.36 | 0.0787 | 1.87831 | 9.41 | 0.0945 |
| 52.4314 | 57.20 | 0.0720 | 1.74374 | 10.69 | 0.0864 |
| 54.8348 | 69.92 | 0.0787 | 1.67424 | 13.07 | 0.0945 |
| 55.5396 | 11.59 | 0.1968 | 1.65465 | 2.17 | 0.2362 |
| 55.9651 | 23.70 | 0.0787 | 1.64308 | 4.43 | 0.0945 |
| 57.0229 | 12.34 | 0.3149 | 1.61509 | 2.31 | 0.3779 |
| 59.0808 | 20.23 | 0.0787 | 1.56365 | 3.78 | 0.0945 |
| 61.5328 | 12.98 | 0.1181 | 1.50709 | 2.43 | 0.1417 |
| 62.4572 | 6.28 | 0.2362 | 1.48699 | 1.17 | 0.2834 |
| 63.6881 | 7.14 | 0.2362 | 1.46119 | 1.33 | 0.2834 |
| 67.0038 | 4.93 | 0.2362 | 1.39672 | 0.92 | 0.2834 |
| 68.6741 | 8.43 | 0.4723 | 1.36676 | 1.58 | 0.5668 |
| 69.8257 | 7.55 | 0.3149 | 1.34702 | 1.41 | 0.3779 |
| 71.4605 | 25.78 | 0.1181 | 1.32017 | 4.82 | 0.1417 |
| 74.4491 | 40.54 | 0.0720 | 1.27335 | 7.58 | 0.0864 |
| 82.2429 | 22.18 | 0.1181 | 1.17225 | 4.15 | 0.1417 |
| 86.5543 | 12.19 | 0.1440 | 1.12366 | 2.28 | 0.1728 |

**Phytochemical Analysis**

**Table S6**. The results of the phytochemical analysis of the investigated extracted samples.

| Samples | Phenolic Content ^[a]^ | Flavonoid Content ^[b]^ | Tannin Content ^[c]^ |
| --- | --- | --- | --- |
| *N. graveolens* extract | 111.2±0.27 | 65.8±0.16 | 16.39±0.09 |
| Ag/CeO_2_ NC | 62.725±0.81 | 43.20±0.34 | 12.14±0.34 |
| Ag/Bi_2_O_3_ NC | 64.639±0.95 | 47.37±1.06 | 13.5±0.81 |

**^[a]^** Phenolic Content “mg gallic acid/1 gm dry extract”

**^[b]^** Flavonoid Content “mg catechin acid/1 gm dry extract”

**^[c]^** Tannins Contents “mg tannic acid acid/1 gm dry extract”

**Antioxidant Activity**

**Table S7**. The Antioxidant Results by DPPH assay.

| Samples | Concentrations (mg/mL) | % Remaining DPPH | % Scavenging Activity | IC_50_ (mg/mL) |
| --- | --- | --- | --- | --- |
| *N. graveolens* extract | 0.21 | 6.754±1.06 | 93.25±1.06 | 0.067±0.72 |
|  | 0.11 | 46.08±0.53 | 53.92±0.53 |  |
|  | 0.05 | 57.84±0.82 | 42.16±0.82 |  |
|  | 0.03 | 67.43±0.47 | 32.57±0.47 |  |
| Ag/CeO_2_ NC | 2.28 | 28.0±1.30 | 72.0±1.30 | 1.073±0.77 |
|  | 1.14 | 46.95±0.77 | 53.05±0.77 |  |
|  | 0.57 | 66.45±0.19 | 33.55±0.19 |  |
|  | 0.29 | 72.22±0.80 | 27.78±0.80 |  |
| Ag/Bi_2_O_3_ NC | 1.52 | 40.52±0.04 | 59.48±0.04 | 1.068±0.60 |
|  | 0.76 | 55.45±0.39 | 44.55±0.39 |  |
|  | 0.38 | 72.33±0.94 | 27.67±0.94 |  |
|  | 0.19 | 78.32±1.03 | 21.68±1.03 |  |
| Ascorbic acid | 0.06 | 15.27±1.45 | 84.73±1.45 | 0.022±0.48 |
|  | 0.03 | 39.08±0.16 | 60.92±0.16 |  |
|  | 0.02 | 61.07±0.09 | 38.93±0.09 |  |
|  | 0.01 | 74.81±0.23 | 25.19±0.23 |  |

**Figure S3**. A relationship plotted the sample concentration (mg/mL) versus % remaining DPPH.

**Ferric Reducing Power (FRAP) Assay**

**Table S8**. Antioxidant results by Ferric Reducing Power Assay.

| Sample | Concentration (mg/mL) | Absorbance read |
| --- | --- | --- |
| *N. graveolens* extract | 6.78 | 0.933 |
| Ag/CeO_2_ NC | 6.78 | 0.485 |
| Ag/Bi_2_O_3_ NC | 6.78 | 0.506 |

**Antibacterial Activity**

**Table S9**. The antibacterial results against various pathogenic bacteria.

| Microorganisms | Inhibition zones in mm (mean value ± SD) | | | |
| --- | --- | --- | --- | --- |
| *Gram-negative bacteria* | ***N. graveolens*** | **Ag/CeO_2_ NC** | **Ag/Bi_2_O_3_ NC** | **Azithromycin** |
| *Escherichia coli* (ATCC 10536) | NA | 12.0 ± 1.50 | 12.0 ± 1.42 | 18.0 ± 1.27 |
| *Salmonella typhimurium* (ATCC 25566) | NA | 14.0 ± 1.43 | NA | 13.0 ± 1.34 |
| *Klebsiella pneumonia* (ATCC 10031) | NA | 13.0 ± 1.94 | 13.0 ± 1.19 | 19.0 ± 1.51 |
| *Enterobacter cloacae* (DMS 30054) | NA | 12.0 ± 1.52 | 12.0 ± 1.38 | NA |
| *Gram-positive bacteria* |  |  |  |  |
| *Bacillus subtilis* (DMS 1088) | NA | 12.0 ± 2.01 | 11.0 ± 1.71 | 22.0 ± 1.27 |
| *Bacillus cereus* (EMCC number 1080) | NA | 12.0 ± 1.44 | 12.0 ± 1.50 | 14.0 ± 1.01 |
| *Staphylococcus aureus* (ATCC 6538) | NA | 17.0 ± 1.83 | 18.0 ± 1.76 | 25.0 ± 1.30 |
| *Staphylococcus epidermidis* (EMCC number 1353^t^) | NA | 14.0 ± 1.77 | 15.0 ± 1.16 | 21.0 ± 1.42 |

NA: refers to no activity.

The experiments were run in triplicate, and the results are expressed as mean value ± standard deviation ((SD).

**Figure S4**. Antibacterial activity of Ag/CeO_2_, and Ag/Bi_2_O_3_ nanocomposites at 6.78 mg/mL in comparison with Azithromycin against various pathogenic bacteria.

**Insecticidal Activity**

**Table S10.** Toxicity of *N. graveolens* extract and nanocomposites on *Aphis craccivora* after one day under laboratory conditions.

| **Treatments** | **Conc**  **(ppm)** | **Insecticidal activity after 24 hours of treatment** | | | | | | | | | |
| --- | --- | --- | --- | --- | --- | --- | --- | --- | --- | --- | --- |
|  |  | **Mortality %** | **LC_50_ (ppm)** | **Confidence limit (ppm)** | | **LC_90_ (ppm)** | **Confidence limit (ppm)** | | ***Slope***  ***± S.E.*** | ***R*** | **Toxicity Index (%)** |
|  |  |  |  | **Lower** | **Upper** |  | **Lower** | **Upper** |  |  |  |
| *N. graveolens* | 50 | 22.1 ± 2.4^E^ | 165.2 | 150.8 | 179.6 | 325.4 | 295.1 | 355.7 | 2.45±0.21 | 0.992 | 0.17 |
|  | 100 | 34.5 ± 2.9^D^ |  |  |  |  |  |  |  |  |  |
|  | 150 | 47.3 ± 3.1^C^ |  |  |  |  |  |  |  |  |  |
|  | 200 | 60.8 ± 3.5^B^ |  |  |  |  |  |  |  |  |  |
|  | 250 | 71.6 ± 3.8^A^ |  |  |  |  |  |  |  |  |  |
|  | *LSD_0.05_* | 5.4 |  |  |  |  |  |  |  |  |  |
|  | *P value* | <0.001*** |  |  |  |  |  |  |  |  |  |
| Ag/CeO_2_ NC | 5 | 31.4 ± 2.5^E^ | 18.7 | 16.20 | 21.23 | 42.36 | 37.82 | 46.87 | 3.12±0.28 | 0.987 | 1.55 |
|  | 10 | 46.7 ± 2.7^D^ |  |  |  |  |  |  |  |  |  |
|  | 25 | 66.5 ± 3.0^C^ |  |  |  |  |  |  |  |  |  |
|  | 50 | 81.3 ± 3.2^B^ |  |  |  |  |  |  |  |  |  |
|  | 100 | 92.4 ± 2.1^A^ |  |  |  |  |  |  |  |  |  |
|  | *LSD_0.05_* | 4.7 |  |  |  |  |  |  |  |  |  |
|  | *P value* | 0.001*** |  |  |  |  |  |  |  |  |  |
| Ag/Bi_2_O_3_ NC | 5 | 25.3 ± 2.2^E^ | 22.45 | 19.87 | 25.31 | 51.63 | 46.21 | 57.82 | 2.89±0.25 | 0.991 | 1.29 |
|  | 10 | 39.2 ± 2.6^D^ |  |  |  |  |  |  |  |  |  |
|  | 25 | 59.8 ± 3.3^C^ |  |  |  |  |  |  |  |  |  |
|  | 50 | 74.6 ± 3.1^B^ |  |  |  |  |  |  |  |  |  |
|  | 100 | 86.1 ± 2.9^A^ |  |  |  |  |  |  |  |  |  |
|  | *LSD_0.05_* | 5.1 |  |  |  |  |  |  |  |  |  |
|  | *P value* | <0.001*** |  |  |  |  |  |  |  |  |  |
| Azadirachtin (Okios 3.2% EC) | 5 | 19.6 ± 2.0^E^ | 28.94 | 25.32 | 32.55 | 68.51 | 61.43 | 75.66 | 2.67±0.30 | 0.989 | 100 |
|  | 10 | 33.1 ± 2.4^D^ |  |  |  |  |  |  |  |  |  |
|  | 25 | 52.4 ± 2.8^C^ |  |  |  |  |  |  |  |  |  |
|  | 50 | 68.2 ± 3.0^B^ |  |  |  |  |  |  |  |  |  |
|  | 100 | 80.7 ± 3.3^A^ |  |  |  |  |  |  |  |  |  |
|  | *LSD_0.05_* | 4.9 |  |  |  |  |  |  |  |  |  |
|  | *P value* | 0.002** |  |  |  |  |  |  |  |  |  |

Mortality was expressed as mean ± SE (standard error) of 3 replicates. LC_50_; LC_90_; upper limit; lower limit their confidence limits at 95%. Different letter per each treatment mean values significance at probability level of 0.05. *** p < 0.001.

**Table S11.** Toxicity of *N. graveolens extract,* nanocomposites, and azadirachtin on *Brevicoryne brassicae* after one day under laboratory conditions.

| **Treatments** | **Conc**  **(ppm)** | **Insecticidal activity after 24 hours of treatment** | | | | | | | | | | |
| --- | --- | --- | --- | --- | --- | --- | --- | --- | --- | --- | --- | --- |
|  |  | **Mortality %** | **LC_50_ (ppm)** | **Confidence limit (ppm)** | | **LC_90_ (ppm)** | **Confidence limit (ppm)** | | ***Slope***  ***± S.E.*** | **R** | **Toxicity Index (%)** |  |
|  |  |  |  | **Lower** | **Upper** |  | **Lower** | **Upper** |  |  |  |  |
| *N. graveolens* | 50 | 20.5 ± 2.1 | 158.34 | 145.24 | 171.63 | 310.75 | 282.44 | 339.07 | 2.41±0.19 | 0.992 | 0.48 |  |
|  | 100 | 32.8 ± 2.4 |  |  |  |  |  |  |  |  |  |  |
|  | 150 | 46.2 ± 2.9 |  |  |  |  |  |  |  |  |  |  |
|  | 200 | 59.7 ± 3.3 |  |  |  |  |  |  |  |  |  |  |
|  | 250 | 70.4 ± 3.6 |  |  |  |  |  |  |  |  |  |  |
|  | *LSD_0.05_* | 5.32 |  |  |  |  |  |  |  |  |  |  |
|  | *P value* | <0.001*** |  |  |  |  |  |  |  |  |  |  |
| Ag/CeO_2_ NC | 5 | 28.3 ± 2.2 | 15.82 | 13.51 | 18.16 | 38.27 | 34.11 | 42.38 | 3.08±0.25 | 0.989 | 4.72 |  |
|  | 10 | 42.6 ± 2.7 |  |  |  |  |  |  |  |  |  |  |
|  | 25 | 63.9 ± 3.1 |  |  |  |  |  |  |  |  |  |  |
|  | 50 | 79.5 ± 3.4 |  |  |  |  |  |  |  |  |  |  |
|  | 100 | 91.8 ± 2.3 |  |  |  |  |  |  |  |  |  |  |
|  | *LSD_0.05_* | 4.95 |  |  |  |  |  |  |  |  |  |  |
|  | *P value* | <0.001*** |  |  |  |  |  |  |  |  |  |  |
| Ag/Bi_2_O_3_ NC | 5 | 24.1 ± 2.0 | 19.41 | 16.83 | 22.06 | 46.53 | 41.76 | 51.34 | 2.95±0.23 | 0.991 | 3.84 |  |
|  | 10 | 38.4 ± 2.5 |  |  |  |  |  |  |  |  |  |  |
|  | 25 | 58.2 ± 3.0 |  |  |  |  |  |  |  |  |  |  |
|  | 50 | 73.6 ± 3.2 |  |  |  |  |  |  |  |  |  |  |
|  | 100 | 85.3 ± 2.9 |  |  |  |  |  |  |  |  |  |  |
|  | *LSD_0.05_* | 5.13 |  |  |  |  |  |  |  |  |  |  |
|  | *P value* | <0.001*** |  |  |  |  |  |  |  |  |  |  |
| Azadirachtin (Okios 3.2% EC) | 5 | 16.8 ± 1.9 | 74.63 | 68.22 | 81.09 | 182.45 | 165.36 | 199.58 | 2.58±0.21 | 0.987 | 1.00 |  |
|  | 10 | 30.2 ± 2.3 |  |  |  |  |  |  |  |  |  |  |
|  | 25 | 51.7 ± 2.8 |  |  |  |  |  |  |  |  |  |  |
|  | 50 | 66.3 ± 3.0 |  |  |  |  |  |  |  |  |  |  |
|  | 100 | 78.9 ± 3.3 |  |  |  |  |  |  |  |  |  |  |
|  | *LSD_0.05_* | 4.71 |  |  |  |  |  |  |  |  |  |  |
|  | *P value* | 0.002* |  |  |  |  |  |  |  |  |  |  |

Mortality was expressed as mean ± SE (standard error) of 3 replicates. LC_50_; LC_90_; upper limit; lower limit their confidence limits at 95%. Different letter per each treatment mean values significance at probability level of 0.05. *** p < 0.001.

**References**

1. de Dobbeleer, I., Gummersbach, J., Huebschmann, H.-J., Mayer, A. & Silcock, P. Analyzing PBDEs in house dust samples with the Thermo Scientific TSQ Quantum XLS Ultra GC-MS/MS in EI-SRM mode. Thermo Fisher Scientific, 1-6 (2012).

2. Elattar, K. M. *et al.* Multifaceted chemical and bioactive features of Ag@TiO_2_ and Ag@SeO_2_ core/shell nanoparticles biosynthesized using *Beta vulgaris* L. extract. *Heliyon* **10**, e28359 (2024).

3. Sánchez-Rangel, J. C., Benavides, J., Heredia, J. B., Cisneros-Zevallos, L. & Jacobo-Velázquez, D. A. The Folin-Ciocalteu assay revisited: improvement of its specificity for total phenolic content determination. *Anal. Methods* **5**, 5990-5999 (2013).

4. Shraim, A. M., Ahmed, T. A., Rahman, M. M. & Hijji, Y. M. Determination of total flavonoid content by aluminum chloride assay: a critical evaluation. *LWT* **150**, 111932 (2021).

5. Elattar, K. M. *et al.* Melanin synthesized by the endophytic *Aureobasidium pullulans* AKW: a multifaceted biomolecule with antioxidant, wound healing, and selective anti-cancer activity. *Curr. Top. Med. Chem.* **24**, 2141-2160 (2024).

6. Vijayalakshmi, M. & Ruckmani, K. Ferric reducing antioxidant power assay in plant extract. *Bangladesh J. Pharmacol.* **11**, 570-572 (2016).
